# Overexploitation risks disproportionate vertebrate downsizing and declines in potential dispersal distances in frugivory networks

**DOI:** 10.1098/rspb.2026.0578

**Published:** 2026-10-28

**Authors:** Oscar Morton, Miguel Lurgi, David P. Edwards

**Affiliations:** ^1^ School of Biosciences, The University of Sheffield, Sheffield, UK; ^2^ Department of Plant Sciences, University of Cambridge, Cambridge, UK; ^3^ Conservation Research Institute, University of Cambridge, Cambridge, UK; ^4^ Department of Biosciences, Swansea University, Swansea, UK; ^5^ Theoretical Sciences Visiting Program, Okinawa Institute of Science and Technology Graduate University, Onna, Japan

**Keywords:** overexploitation, seed dispersal, frugivory networks, defaunation, cascading effects, wildlife trade, biodiversity conservation, ecosystem function

## Abstract

Poorly managed and unsustainable exploitation from either the use or trade of species can be a key driver of wildlife population declines and extirpation risk. Local extirpation of overexploited vertebrate species risks triggering cascading ecosystem effects through plant–frugivore interactions, disrupting the ecological networks they form, with potential impacts on the associated seed-dispersal services. Using a global database of the occurrence of more than 16 000 reported bird and mammal frugivory interactions across 160 binary frugivory networks containing likely overexploited species, we simulated the extirpation of overexploited species and their cascading impacts on network composition and potential ecosystem function. The loss of overexploited species triggered a sixfold greater body mass downsizing of vertebrate communities than would be expected under random species loss. By contrast, plant communities experienced comparable structural disruption in frugivory networks following overexploitation-driven versus random loss. Average seed-dispersal distances within networks declined sharply following the extirpation of overexploited species. Average distances declined 161% more after the extirpation of overexploited species relative to the equivalent randomized species loss (12.0% decline after overexploitation, 4.6% after random loss). Our results demonstrate that overexploited species have a key role in local frugivory networks, underscoring the importance of maintaining ecosystem integrity via the protection of vertebrate populations to restore ecosystem function in degraded or defaunated landscapes.

## 1. Introduction

The hunting and use of vertebrates worldwide provides key nutrition for local communities and key income for conservation activities [1,2]. However, where poorly managed, exploitation for subsistence or commerce is a key driver of population declines and the wider biodiversity crisis [3–5]. In some local ecosystems, intensive defaunation pressure has driven the local extirpation of specific components of the original faunal community (e.g. some larger-bodied species), even in relatively intact habitats, leaving ‘*empty*’ or ‘*half-empty*’ ecosystems [6,7]. Beyond the intrinsic local loss of these exploited species, there is the potential loss of key functional roles performed by these species and of the associated ecosystem services. One of these key services potentially at risk is the dispersal of seeds by vertebrate species, key for plant reproduction [8–10]. These secondary effects can cascade locally to impact ecosystem-wide ecological processes, including recruitment and regrowth of plant species [11,12]. Given the vast diversity of vertebrate species used by humans, the broader ecological footprint (i.e. beyond biodiversity loss) of poorly managed or unsustainable use risks being substantial [13,14].

Up to 75% of tree species in the tropics and 60% in temperate regions rely on mobile animals for seed dispersal [15]. Yet this varies broadly, with animal-mediated dispersal especially key across typically more modular and specialized neotropical and Asian lowland forests [16]. Across local ecosystems, the loss or depressed abundance of exploited bird and mammal species is hypothesized to trigger cascading impacts among the local plant community. This includes the secondary extirpation of plants reliant on specific dispersers. However, evidence supporting this hypothesis remains divided [17–19]. Nevertheless, altered patterns of seed dispersal function are well documented and suggest that defaunation triggers decreased dispersal distances, increased spatial clustering of seeds, decreased removal of larger seeds, the loss of evolutionarily distinct interactions and the subsequent homogenization of plant communities [20–23]. Furthermore, modelling studies suggest that these impacts can subsequently affect how those communities respond to co-occurring threats, for instance, hampering plants’ ability to track climate change and driving synergistic biomass losses when coupled with increasing fire frequency [24,25]. While direct field studies and manipulation experiments demonstrate a breakdown in seed-dispersal interactions post-defaunation [26–28], theoretical models go further and suggest that such breakdowns can disrupt patterns of carbon storage and sequestration. Carbon losses are projected to be particularly acute in African, South American and South Asian forests [29–31].

Obtaining a better understanding of the effects of overexploitation on ecosystem services has focused predominantly on the loss of large-bodied bird and mammal species and subsequent impacts on the wider structure and functioning of seed dispersal networks [21,30,32]. However, the globalization of exploitation and commercial trade, coupled with rapid shifts in the species experiencing high demand, has resulted in a diverse array of species threatened by overexploitation [14,33–35]. This is exemplified by the ongoing Asian songbird crisis, where many smaller-bodied songbird species (including Bali myna *Leucopsar rothschildi*, straw-headed bulbul *Pycnonotus zeylanicus* and Sumatran laughingthrush *Garrulax bicolor*) have experienced severe population declines across their range [36]. Through frugivory, many of these species play critical roles in local seed dispersal (e.g. bulbuls) [37]. This is in addition to the exploitative pressure that large-bodied seed dispersers face globally, such as the helmeted hornbill (*Rhinoplax vigil*), traded predominantly for its ‘red ivory’ casque [38] and *Tapirus* sp. hunted for consumption [39].

A key gap in knowledge is understanding how overexploited species specifically interact within their local ecosystem and how the loss of the species most likely to be locally extirpated disproportionately disrupts each level of the frugivory process (from the vertebrate community all the way to resulting dispersal function). To address this question, we leverage an extensive global database of the occurrence of more than 16 000 distinct field-verified binary plant–frugivore (bird and mammal) interactions [40], coupled with comprehensive predictions of plant-vertebrate-specific seed-dispersal distances [24] and assessments of overexploitation pressure for vertebrates [41]. The combination of these datasets allows us to model the local losses of overexploited species and assess how they alter local vertebrate communities within the interaction network, how these effects cascade through to the plant communities, and ultimately to potential seed-dispersal functioning, enabling an assessment of where in this hierarchy impacts manifest. We ask three specific questions: (i) does the simulated extirpation of overexploited species drive disproportionate impacts on the vertebrate community via altered average diet breadth or mass downsizing, (ii) does this trigger structural impacts on the plant community owing to altered disperser richness per species or secondary structural extirpations, and (iii) does the simulated extirpation of overexploited species risk disproportionate impacts on potential seed-dispersal functioning via altered dispersal distances?

## 2. Methods

### (a) Data collation

We used a recently published database of 396 plant–frugivore networks [40] encompassing 30 892 unique pairwise interactions between 1636 animals (birds, mammals, squamates and testudines) and 3209 plant species, including associated metadata (e.g. latitude and longitude) drawn from 241 field studies (supplementary material, figure S1). The binary plant–frugivore interaction data includes the presence of all reported interactions between vertebrates and fleshy-fruited plants at the study sites (see [40] for details on the data collection process). Birds and mammals represent 99.4% of vertebrates in these networks and are the focus of this study. We note, however, that while this data represents one of the largest compilations of frugivory interactions to date, it lacks abundance or interaction-specific weighting. Thus, there is a risk that such binary interaction networks may understate the importance of smaller, abundant species and overinflate the role of larger, rarer species, as it essentially assumes that each interaction occurs equally frequently and contributes equally to ecological processes (e.g. ingests the same amount of fruit). However, standardized interaction-abundance data do not exist at the scale required here. Despite this, unweighted links still allow us to rigorously map the underlying architecture of the community, independent of population sizes. We complemented these data with avian and mammalian body mass data from two comprehensive databases, AVONET and PHYLACINE [42,43].

We reconciled taxonomies to align with the latest version of the International Union for Conservation of Nature (IUCN) Red List and extracted species' latest entire assessments using the Red List API v. 4, accessed using the package ‘*rredlist*’ [44]. To understand which species are potentially at risk of local overexploitation, rather than simply being used or traded, we used the species-specific IUCN threat tables. From these, we extracted all species recorded as having Biological Resource use–Intentional Use (threat code 5.1.1) as a threat. This particular code captures where the species is the intentional target of some form of harvest or extraction by people and that this exploitation constitutes a threat to the species. We further refined this list by adopting a similar scoring system to Marsh *et al.* [41], scoring species Biological Resource Use threat based on the reported scope, severity and timing of the threat (all scored out of 3), keeping only species that had a cumulative score of at least 6 (supplementary material, table S1). This particular cut-off represents a precautionary approach balancing the potential for uncertainty in the scope, severity and timing of the threat. Out of the 1622 birds and mammals present in the networks, 904 are reported as traded in some form in a recent global assessment [14], 227 had Biological Resource use–Intentional Use listed as a threat, and only 163 reached our scoring criteria to be considered as at risk of overexploitation in at least part of their range. When we refer to species at risk of overexploitation, it is this specific group of 163 species that we refer to and base this assessment on the available IUCN data and established methods [41]. We cross-referenced the occurrence of these 163 species with the frugivory networks, yielding 164 networks containing at least one overexploited species.

We leveraged a recent study modelling the seed-dispersal process as a product of gut passage time, frugivore movement speed and displacement correction (a measure to account for stops and changes in direction to limit dispersal distance) [24]. The study incorporated both vertebrate and plant species traits in addition to effect sizes from published meta-analyses. Their individual models of gut passage time (based on 194 plant and 74 animal species), movement (based on 199 bird and mammal species) and displacement correction (based on 174 plant and 68 animal species) all had good predictive performance values (respectively, *R*^2^ = 0.958, 0.800 and 0.734, but values may be slightly inflated, as cross-validation was also used for model tuning). However, while this remains the best resource for interaction-specific potential seed dispersal distances, it remains trained on a sample of observed interactions and does not explicitly account for potentially influential broad additional factors, such as phylogenetic signal in dispersal or spatial variation within specific interactions. Building larger field-based estimates of interaction-specific seed dispersal remains a key research frontier. The output of this was a database of predicted species-interaction-specific (vertebrate–plant) dispersal distances. We matched the vertebrate–plant interactions across the 164 networks with the predicted interaction-specific dispersal distances. We dropped networks where we had less than 75% coverage across the network for estimated dispersal distances among taxa identified to the species level, leading to the removal of four networks. Across the remaining 160 networks, the mean coverage of dispersal distance across interactions was 98.8%, lending confidence in the accuracy of information completeness across these networks.

### (b) Simulating local extirpation

We used six metrics to assess the impact of simulated vertebrate species extirpation on networks, two focused on the vertebrate community, two on the plant community and two on the potential seed dispersal outcome. All metrics were calculated for the intact networks and after simulating extirpation. All metrics were scaled relative to the intact network's value.

— Mean frugivore diet breadth: defined here as the number of unique plant species each extant frugivore consumes, averaged across the local network. Higher values are associated with generalist vertebrate species with the potential to disperse a greater diversity of plant species. Depending on the species extirpated from the network, this value can decrease or increase above 100% of the intact community.
— Mean vertebrate community body mass: defined as the mean body mass of the extant vertebrate community. Decreasing values would be indicative of community downsizing, a common phenomenon in certain hunted ecosystems. Depending on the species extirpated from the local networks, this value can decrease or increase above 100% of the intact community.
— Mean number of dispersers per plant species: defined as the number of extant vertebrates interacting with each plant species, averaged across the network. Higher values are associated with generalist plant species with a propensity to be dispersed by a wide range of animal species. The extirpation of vertebrate species will always cause this value to decline to less than the intact network.
— Number of plant secondary extirpations: we assume plant species are locally extirpated when no extant vertebrate species known to interact with that plant species remain in the network [45]. Our approach is conservative as it considers secondary extirpations only after complete structural isolation. We summarize this value as the percentage of the intact plant community that is secondarily extirpated; thus, this value ranges from 0 to 100%. Zero indicates that after vertebrate extirpation, no plants were structurally isolated, and 100 indicates the isolation of all plants (e.g. only possible after the local extirpation of all vertebrates)
— Mean seed-dispersal distance: defined as the mean distance seeds are dispersed across all extant vertebrate–plant interactions in the local network (supplementary material, figure S2). Depending on the species extirpated from the network, this value can decrease or increase above 100% of the intact community.
— Maximum seed-dispersal distance per species: defined as the maximum dispersal distance for each plant species based on the extant vertebrate community, averaged across the network. This metric provides insight into the maintenance or loss of longer-distance dispersal within the frugivory network. The extirpation of species will always cause this value to remain constant or decline to less than the intact network.

For each network, we calculated the intact values for our six metrics and determined the total number of overexploited vertebrate species in the network. Then, we iteratively simulated the extirpation of increasing numbers of overexploited species from the network. For example, if a network contained 10 overexploited species, we first simulated the random loss of one overexploited species and repeated this for all possible combinations of species removal for cases where the total possible number of distinct combinations was smaller than 100; where greater than 100, we took a random sample of 100 possible overexploited species combinations. We calculated the six network-wide metrics per iteration. This procedure was repeated by removing 2, 3, …, *N* randomly sampled overexploited species from the network, again repeating this for all combinations or 100 replicates, until we reached the total number of overexploited species present in the network being extirpated (e.g. in this example of 10 species there are 10 combinations to remove a single species, and 45 combinations to remove 2 species).

We used a null model to contrast the effects of overexploitation-driven loss to that expected by chance. To do this, we simulated the loss of randomly sampled species for each number of overexploited species lost per network, replicated 100 times or for all possible combinations, for each number of species extirpated. We also compared our results with an alternative worst-case null model in which species were iteratively removed from each network by order of decreasing body mass. This represents a highly severe scenario of species loss, far more extreme than the loss of likely overexploited species. While larger-bodied species are more likely to be hunted and threatened [6], the vast majority of species are not explicitly likely to be threatened by overexploitation (per the IUCN-based metrics detailed above). Thus, many larger-bodied species (even if they are hunted or used) are not explicitly threatened by overexploitation. Across the frugivory networks studied, the median ratio of exploited to unexploited species’ body mass was 13.7 (although this was primarily driven by a small number of outlying networks containing a single very large species, such as *Loxodonta africana*, the median ratio of the lowest 80% of the data was 1.9, in figure S3 in the supplementary material*.* The potential impacts of completely size-structured extirpations are shown in figures S4–S6 in the supplementary material.

This yielded 100 values per metric, per network, per number of species removed (up to the total number of overexploited species per network) for both overexploited and randomly selected species, for which we calculated the average of each metric. The raw numbers of species extirpated per network were converted to percentages of the intact network richness to facilitate comparisons across networks of differing sizes. For each metric, we built Bayesian generalized additive models with a Gaussian error distribution, modelling each of the six metrics as a function of the extirpation scenario (random loss or overexploited species only) and the proportion of species loss with separately penalized thin-plate splines with a basis dimension of 5 per extirpation scenario (increasing this triggered no clear change in model fit or inference). We additionally included hierarchical intercepts for network ID to reflect that the scenarios and proportion of species lost are nested within networks.

All models were fit with ‘*brms’* [46] using diffuse, zero-centred priors (see supplementary material, table S2 for full details). Models were run for 800 warm-up iterations and 800 sampling iterations across four parallel chains. Stable convergence was assessed in all models using the Rhat statistic (all values less than 1.01, see supplementary material, table S3 for further details). Model fit and adequacy were assessed using posterior predictive plots, which showed excellent recovery of observed properties (supplementary material, figure S7). All model outputs were summarized by the 95% highest density interval (HDI) and the median. Comparisons and differences were all calculated using the full posterior distribution (supplementary material, figures S8–S10). All data handling and model processing were undertaken in R v. 4.4.2 [47], using ‘*tidyverse’* [48] and ‘*tidybayes’* [49].

### (c) Sensitivity analyses

We carried out two forms of sensitivity analysis. Firstly, while we only consider the local extirpation of species where overexploitation is a likely threat in at least part of their range, this does include some species assessed as least concern. While the extinction of such species is unlikely, we do consider the local decline and potential extirpation of these species plausible. Historically abundant species have undergone rapid population declines owing to multiple concerted threats, for example, the now critically endangered straw-headed bulbul (*P. zeylanicus*), whose populations are estimated to have declined by greater than 80% over the last 15 years [50], and the African forest elephant (*Loxodonta cyclotis*), which now occupies less than 25% of its potential range [51]. However, as their local extirpation is considerably less likely than for more threatened species, we repeated the analyses presented in the main text after assuming all least concern species cannot become locally overexploited. This reduced the pool of likely overexploited species to 107 and the number of local networks containing these species to 54. Results from these analyses are presented in full in figures S11–S13 in the supplementary material and align closely with the results presented in the main text.

Secondly, biases and a lack of comprehensive network sampling in the original field studies may potentially bias these results. We minimize these biases by focusing on relative differences within local networks before and after simulated overexploitation, rather than between network comparisons. Prior studies using this dataset have found highly comparable results after focusing only on networks from studies that self-reported having comprehensively recorded all interactions for both plants and the target vertebrate taxa, rather than just those for a subset of species [40]. In addition to focusing on within-local network comparisons, we do the same and repeat in full the analyses presented in figures 1–3, but only considering networks reported as comprehensively sampled, which reduced the sample of networks from 160 to 99. The results from these analyses are presented in full in figures S14–S16 in the supplementary material and, as above, align closely with the main text results.

## 3. Results

Species likely at risk of overexploitation (defined using the IUCN Red List as species with a high cumulative scope, severity and timing score for threat from intentional use) occurred in 160 plant–frugivore binary interaction networks collated in a recent global effort [24] (figure 1a). The majority of frugivory occurrence networks containing overexploited species had a single overexploited species, with only 19 networks having five or more species at risk of overexploitation, all of which were located in the tropics (see supplementary material, table S4 for a detailed breakdown of network coverage). Likewise, the proportion of likely overexploited vertebrates per network ranged from 0.01 to one (supplementary material, table S4). These network locations overlap with known hot spots of traded diversity and overexploitation risk, particularly across South American hot spots in the Amazon basin and Andes and across Indochina [6,14]. However, sub-Saharan Africa, where defaunation pressure is known to be high, had relatively poor network coverage [6]. European networks were always represented by no more than a single species at risk of overexploitation.

**Figure 1.**
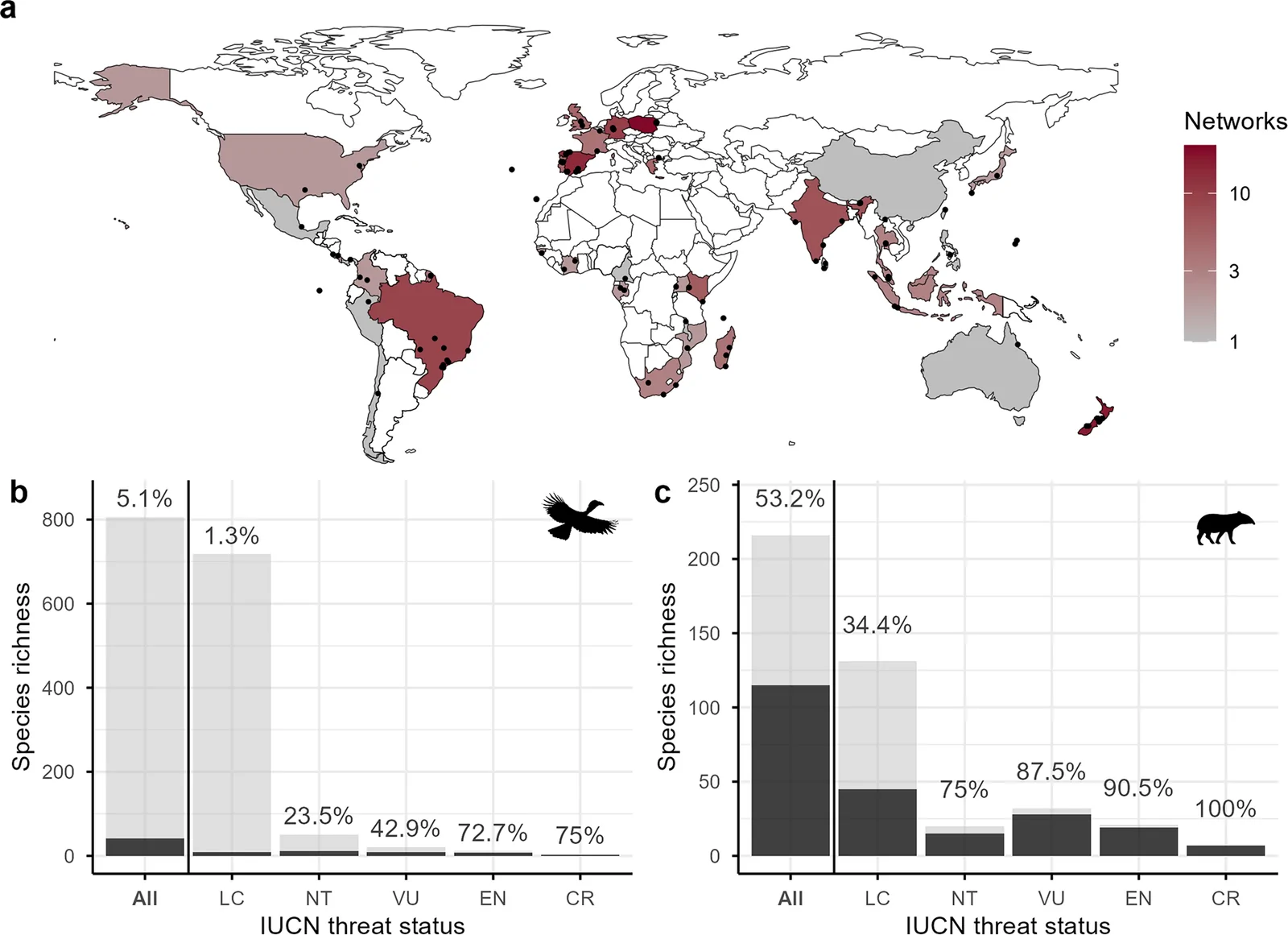
Global distribution of the diversity of traded species across plant–frugivore interaction networks. (a) Locations of all plant–frugivore networks that contain at least one overexploited species (*n* = 160). Countries are coloured by the number of these networks (see supplementary material, figure S1 for a map of all networks in the original dataset). (b,c) Coverage across IUCN Red List categories for (b) birds and (c) mammals. Red List categories are as follows: LC, least Concern; NT, near threatened; VU, vulnerable; EN, endangered and CR, critically endangered. All denotes total species richness across all categories. Black sections of bars show overexploited species richness (shown as a percentage above each bar). Grey sections denote non-overexploited species. Shown for the 160 networks included in subsequent analyses. Data-deficient and non-evaluated species are not shown. Inset images show *Tapirus* sp. and *Buceros bicornis* sourced from https://www.phylopic.org/images.

Across local plant–frugivore networks, species at risk of overexploitation represented a small fraction of the total diversity, 5.1% of birds and 53.2% of mammals, respectively (163 of the 1022 bird and mammal species recorded in networks containing at least one possibly overexploited species, figure 1b,c). The proportion of species at risk of overexploitation increased with extinction risk with 75.0 and 100% of critically endangered birds and mammals found in the networks classed as at risk of overexploitation. This includes both large- and small-bodied species, including the grey parrot (*Psittacus erithacus*), an endangered large frugivore globally sought after for the international pet trade, and the small-bodied greater green leafbird (*Chloropsis sonnerati*), a highly sought-after songbird. The mean body mass of the exploited mammals present in the networks was 90 053 g, relative to 20 408 g for unexploited species, while for birds it was 1445 and 109 g, respectively.

### (a) Simulated local extirpation of overexploited species triggers downsizing

Overexploited species typically made up a small proportion of network vertebrate diversity, with only 20% of networks comprising greater than 30% likely overexploited species (figure 2a). Thus, looking beyond the loss of 30% of the community quickly refers to increasingly few empirical networks.

**Figure 2.**
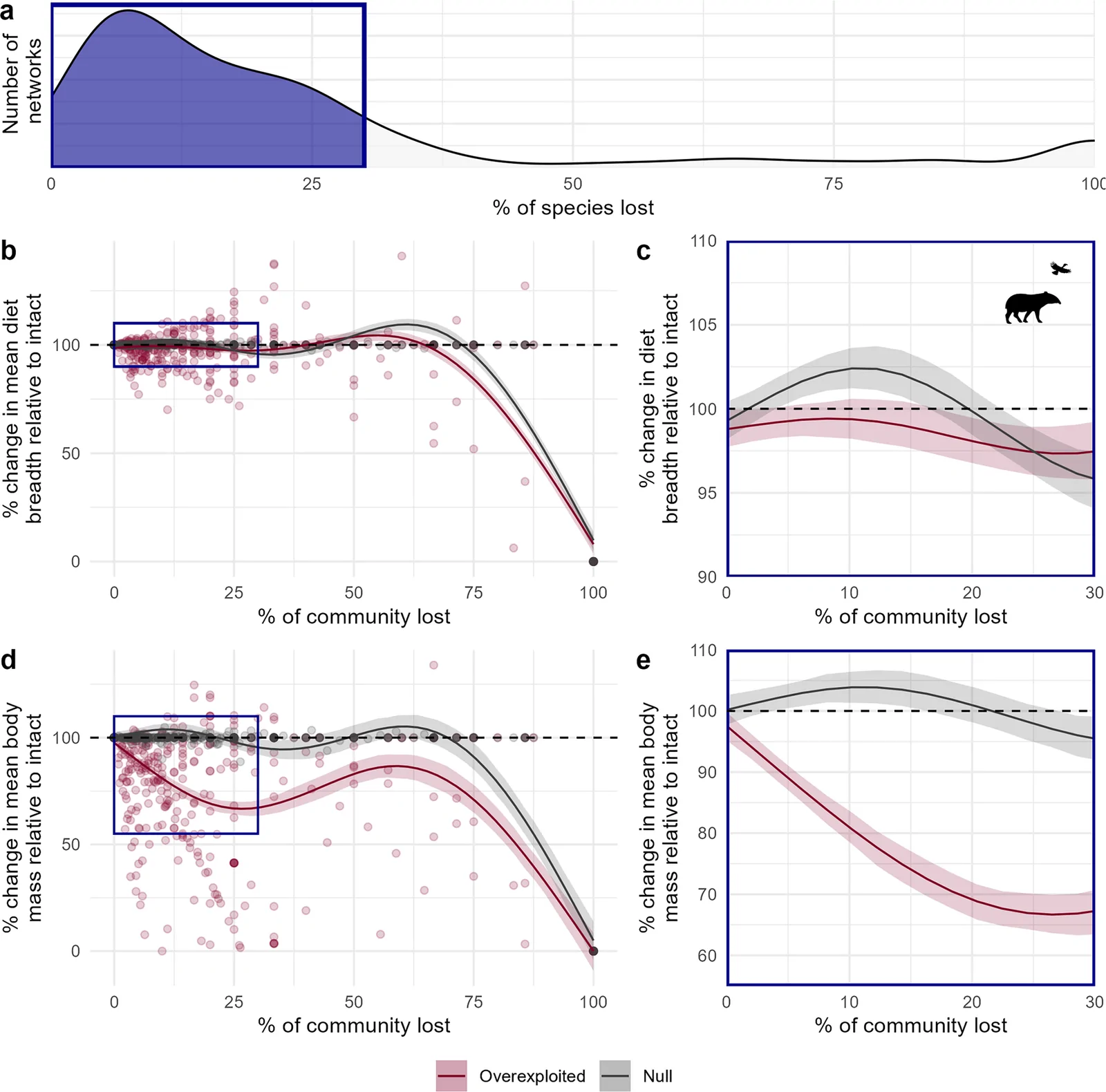
Structural disruption of the vertebrate community following the local loss of likely overexploited species from local frugivory networks. (a) The distribution of exploited species proportionate presence in 160 frugivory networks, blue bounding box shows that 80% of all networks contain 30% or fewer overexploited species. (b) Percentage change in mean community frugivore diet breadth after the loss of overexploited (red) and randomly sampled (black) species, the blue bounding box highlights losses up to 30% of the intact community richness (e.g. the loss of 30% of vertebrates present in the intact network), which is enlarged for ease of visualization in (c). (d,e) Show the equivalent information for the change in community average body mass of frugivore species post-extirpation relative to the intact community. Lines are posterior medians and ribbons are the 95% HDI. Each point represents a given network, per sequential extirpation scenario, the null scenario points are the average across either 100 iterations or all possible combinations if fewer than 100. The inset image shows *Tapirus* sp. and *B. bicornis* sourced from https://www.phylopic.org/images and denotes the vertebrate community.

The increasing loss of overexploited species from frugivory networks resulted in very small changes to the average diet breadth of frugivores. This outcome was similar for the random removal scenario (figure 2b,c). Minor differences occur after approximately 10% species loss (a difference of 3.04%, 95% HDI: 1.49–4.57%; supplementary material, figure S8), with the null expectation displaying a very slight increase in diet breadth, compared with no clear change for the equivalent loss of overexploited species.

However, the loss of overexploited species triggered a substantial downsizing effect among the frugivore community (figure 2d). Average community body mass declined to 67.4% (95% HDI: 63.5–70.9%) of the original community after the removal of 30% of the species. This effect is more than six times greater than the equivalent change following random species loss (95.3%, 95% HDI: 91.8–99.0%, figure 2e), yet approximately half of that observed in purely size-structured extirpation scenarios (supplementary material figure S4).

### (b) Limited simulated plant structural changes post-exploitation

Next, we assessed the impact of the extirpation of species likely at risk of overexploitation on the plant community in local frugivory networks. We found that the loss of likely overexploited species was associated with a decline in the richness of potential dispersers per plant species (figure 3a). However, this impact is closely aligned with what would be expected after the random loss of species. The loss of 30% of the intact community triggered a reduction in disperser richness to 69.7% (95% HDI: 68.9–70.6%) and 69.3% (95% HDI: 68.7–70.0%) of the intact community following the loss of overexploited and randomly sampled species, respectively (figure 3b). Similarly, overexploitation-driven and random vertebrate extirpations resulted in highly comparable numbers of risked secondary plant extirpations (figure 3c). The loss of 30% of vertebrate species triggered up to 12.7% (95% HDI: 11.2–14.0%) and 13.1% (95% HDI: 11.7–14.6%) of the plant community to become structurally extirpated (i.e. isolated from the network owing to the loss of all interactions) in overexploited versus random removal, respectively (figure 3d). Only the loss of overexploited species triggered proportionally greater secondary extirpation risks (points above the dashed line, figure 3c), suggesting that in some local networks these species may form key mutualisms with specialized plant species. Simulated size-structured extirpations had similarly comparable impacts to the null and overexploitation-based extirpations (supplementary material, figure S5).

**Figure 3.**
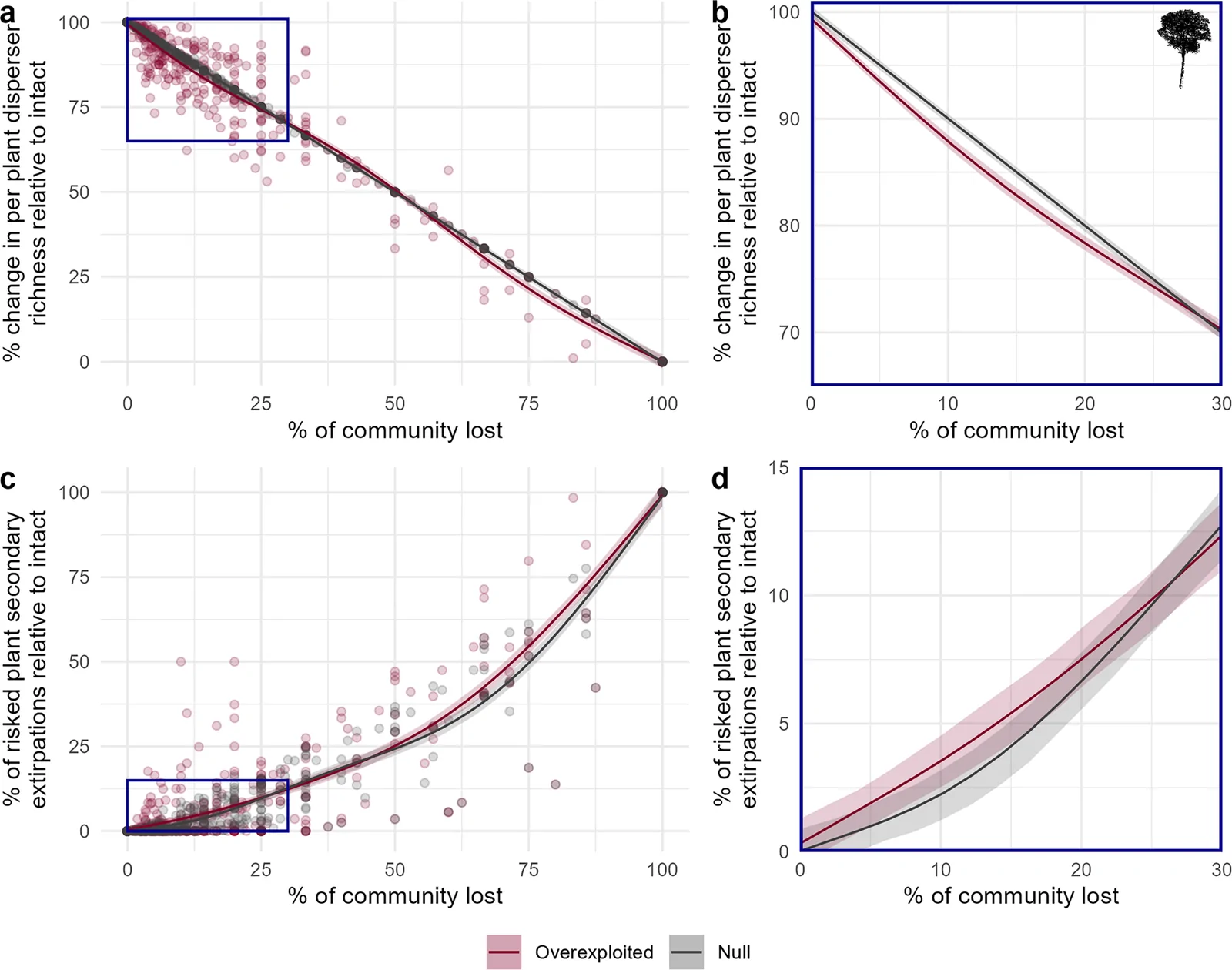
Structural disruption of plant communities following the loss of likely overexploited species from local frugivory networks. (a) Percentage change in mean number of potential dispersers per plant species after the loss of overexploited and randomly sampled species (null expectation). (b) The blue bounding box highlights losses up to 30% of the intact community richness, which is enlarged for ease of visualization. (c,d) Show the same relationship for the number of risked structural secondary extirpations relative to the intact community richness. Lines are posterior medians and ribbons are 95% HDI. Each point represents a given network, per sequential extirpation scenario, the null scenario points are the average across either 100 iterations or all possible combinations if less than 100. The inset image shows *Calophyllum austroindicum* sourced from https://www.phylopic.org/images.

### (c) Overexploited species disproportionately impact seed dispersal potential

To evaluate the effect of losing species likely at risk of overexploitation on the potential functioning of plant–frugivore seed dispersal networks, we assessed the simulated impact of sequential overexploitation-driven extirpations on seed-dispersal distances. Our simulations revealed that increasing the number of local extirpations of overexploited species led to increasing declines in the mean dispersal distance relative to the intact community (figure 4a). The extirpation of likely overexploited species representing up to 30% of the intact vertebrate community triggered a decline in the mean distance that seeds are dispersed across the average local community to 88.0% (85.8–90.5%) of the intact community mean seed dispersal distance, whereas random extirpation only triggered a decline to 95.4% (95% HDI: 92.8–97.7%). On average, this constituted a 161% greater decrease than expected in randomly sampled extirpation scenarios (12.0% decline after overexploitation and 4.6% after random species loss, figure 4b). There was also a disproportionate decrease in the community average maximum dispersal distance per plant species (figure 4c). The random extirpation baseline triggered a decrease to 81.1% (78.9–83.4%) of the intact community's maximum dispersal distance following the loss of up to 30% of the intact community's vertebrate diversity. The equivalent loss of overexploited species triggered a larger decline to 74.1% (71.9–76.5%) of the intact community. A 37% greater decrease than would be expected at random (25.9% decline after overexploitation and 18.9% after random species loss, figure 4d). Notably, despite size-structured extirpations triggering twice as large community downsizing relative to overexploitation-based extirpation scenarios (supplementary material, figure S4), the resulting changes in community maximum dispersal distance were highly comparable (supplementary material, figure S6). Repeating these analyses individually per biogeographical region, the patterns of overexploitation-driven body mass decrease and declining seed-dispersal distance remained relatively homogeneous across the Americas, Africa, Eurasia and Asia Pacific (supplementary material, figures S17–S22).

**Figure 4.**
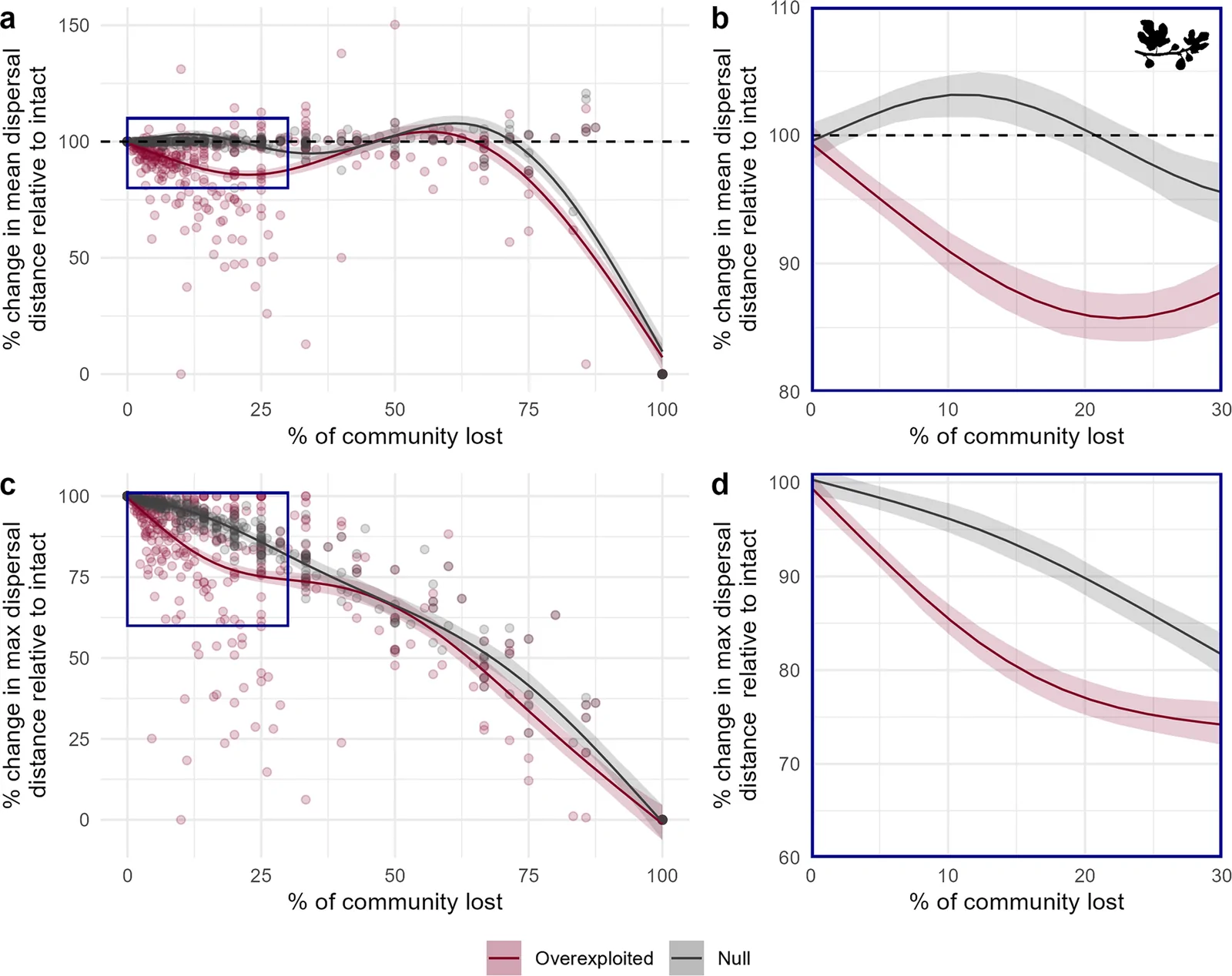
Disruption of seed-dispersal functioning following the loss of likely overexploited species from local frugivory networks. (a) Percentage change in mean dispersal distance per network after the loss of overexploited and randomly sampled species (null expectation), the blue bounding box highlights losses up to 30% of the intact community richness, which is enlarged for ease of visualization in (b). (c,d) Show the same relationship but with the percentage change in the community average maximum dispersal distance per plant species. Lines are posterior medians and ribbons are the 95% HDI. Each point represents a given network, per sequential extirpation scenario, the null scenario points are the average across either 100 iterations or all possible combinations if less than 100. The inset image shows *Ficus carica* fruits sourced from https://www.phylopic.org/images.

## 4. Discussion

Overexploitation is posited as a key threat to plant–frugivore interaction networks with potential repercussions for wider ecosystem functioning. Through simulated local extirpation in frugivory interaction occurrence networks, we demonstrate that the loss of overexploited species risks a disproportionate decrease in the body mass of vertebrate consumers in these binary networks, triggering sixfold greater reductions in mean community body mass than random loss. There was limited evidence that overexploitation disproportionately impacts other aspects of network structural composition (average diet vertebrate breadth, disperser richness per plant or risk of secondary extirpations). However, the simulated extirpation of overexploited species has the potential for a concerted and disproportionate impact on seed-dispersal distance, reducing average and maximum dispersal distances by 161 and 37% more than what would be expected under random loss, respectively.

### (a) Cascading implications of overexploitation

Our simulations suggest that the loss of likely overexploited species could strongly impact vertebrate communities via downsizing and reduced seed dispersal distances (reducing maximum distances by 37% relative to random loss). This reiterates prior work finding that larger birds and mammals are significantly more likely to be threatened by overexploitation [52], and that bird and mammal community biomass often strongly decline along gradients of increasing hunting pressure [53]. The local extirpation of large megafauna probably drives the subsequent reduction in dispersal distance via the loss of larger species whose greater dispersal distances simply cannot be compensated for by smaller frugivores [54]. This lack of compensation can be missed by focusing solely on the structural features of networks rather than on the functional outcomes. In long-lived tropical tree communities, this is especially critical as the loss of dispersal function is more likely to trigger a plant species extinction debt than immediately detectable declines. Populations of long-lived tree species can persist for decades with minimal recruitment before impacts are detectable [55,56]. In frugivory networks across the Andes, simulated downsizing similarly triggered substantial losses of long-distance seed dispersal without triggering increased numbers of immediate secondary extirpations [57]. Our findings align with this form of cascading effects from an overexploitation-driven loss perspective with strong impacts on the vertebrate community but lacking direct effects on the structural aspect of the plant community, instead manifesting indirectly through declining dispersal distances. Field studies have further shown that the loss or decline of larger frugivores can exacerbate seed size selection, increasing seed predation, driving greater kin competition and reducing sapling recruitment [12,58–60]. Theoretical models also suggest these impacts can further cascade to affect ecosystem-wide properties, such as carbon storage [18,29–31]. Something we were unable to consider is the potential for ecological rewiring [61]. Empirical evidence has shown that this rewiring of communities following loss can reduce declines in dispersal distances following extirpation; however, it cannot prevent them fully, and larger-fruited species remain particularly vulnerable [62].

In addition to the loss of larger-bodied species, the loss of threatened species from frugivory networks can also risk a disproportionate ecological impact [19,63]. In the Brazilian Atlantic Forest, the loss of endangered vertebrates or those with declining populations from frugivory networks risked the loss of unique mutualistic frugivory interactions that were not compensated by more abundant species [19]. This interaction deficit, as opposed to compensation, is not uncommon and highlights the critical nature of conserving species interactions in general and mutualistic relationships in particular [20,64–66]. Modelled scenarios of global extirpation of threatened birds and mammals similarly highlight disproportionately large contractions of functional space, particularly among frugivorous mammals threatened by exploitation [67,68]. Thus, as we demonstrate here, the loss of overexploited species risks a form of perfect storm, often capturing both threatened and larger species critical to effective community-wide dispersal.

### (b) Changing patterns of overexploitation

The historic and ongoing extirpation of large mammals for subsistence or commerce has been well documented [12,69,70]. Clear parallels can be drawn with the current, ongoing decline among larger-bodied frugivorous avian taxa, notably the *Bucerotiformes* (hornbills). Across forest landscapes in India, hunting-driven declines in hornbill species abundances have reduced the dispersal of seeds to just a seventh of that in less-disturbed sites [71]. Similar trends are seen in hornbill-dispersed ecosystems in the Democratic Republic of Congo, where seed-dispersal failure increases along a gradient of increasing hunting pressure [72].

Comparing our current understanding of potential hot spots of overexploitation risk [3,5,6] with the current coverage of ecological network data highlights the limits of our ability to identify at-risk biomes and regions, with key regions often poorly represented, e.g. sub-Saharan Africa (supplementary material, figure S1). The congruency in responses in the vertebrate and plant communities and declining seed dispersal distance across the Americas, Africa, Eurasia and Asia Pacific suggests the wider importance of overexploited species in global ecosystems (supplementary material, figures S17–S22). However, field research on intact and disrupted plant–frugivore networks, particularly across Southeast Asian lowland and sub-Saharan African forests, is sorely needed to test this further. This is especially pertinent given the high levels of exploitation across these regions and their concurrent commitments to global restoration efforts [12,36,73–75].

Our use of large-scale datasets engenders several key assumptions. Firstly, there is a risk that for common or abundant species, the threat from overexploitation may be highly spatially restricted to a specific population or region. In almost all cases, the spatial distribution of the intra-specific occurrence of specific threats is poorly quantified. Focusing only on species with a relatively high severity of threat and current timing [41] limits this risk, but cannot entirely remove it. As a precaution, we repeated the main text analyses after removing any least concern species classed as at risk of overexploitation. These results were highly comparable to those presented above, reiterating the reductions in vertebrate body mass, minimal structural impacts on the plant community, but potentially disproportionate losses of seed-dispersal distance after the removal of overexploited species (supplementary material, figures S11–S13). Secondly, a further facet we cannot address at scale is the distinction between local extirpation and local declines; quantifying the exact degree to which species are declining or not, and how these declines change dispersal effectiveness at scale, remains a research frontier.

Thirdly, sampling bias in ecological networks can take many forms, some of which are difficult to determine post-data collection. These biases can include the under-representation of rarer species, rendering them more specialized than in reality [76,77], the more complete sampling of networks in northern latitudes or simply that not all interactions could be observed and thus were not documented. To explore this, we repeated all analyses after removing networks reported to have not comprehensively sampled the target taxa (supplementary material, figures S14–S16). Crucially, these results also closely resemble our main findings, although large swathes of central Africa and Southeast Asia remain poorly represented in the data. Recent work has also highlighted the role that typically non-frugivorous species may have in seed dispersal (e.g. waders) and that these species can be systematically under-represented or absent from ecological network data [78]. However, the extent to which such species can compensate for the loss of more conventional frugivory interactions remains poorly quantified. Lastly, while we used published, species-specific, mechanistic estimates of seed-dispersal distance, these remain modelled estimates and cannot perfectly reflect site-specific behaviourally mediated seed-dispersal distances [79].

### (c) Future directions and conclusions

The loss of overexploited species risks cascading effects through ecosystems via their ecological interactions [11,68,80]. We showed that this manifests as the functional downsizing of vertebrate communities, and this loss of larger-bodied species in turn has the potential to substantially reduce seed-dispersal distances across ecosystems. However, we note limited evidence for structural impacts on plant communities, finding no clear evidence that overexploitation will drive more secondary plant extinctions or losses of mean disperser richness relative to random losses. We echo previous calls for increased consideration of defaunation impacts on forest regeneration prospects and subsequently the potential for forest restoration and protection schemes (e.g. reducing emissions from deforestation and forest degradation in developing countries, REDD+) in exploited landscapes to directly engage in protecting fauna to enhance ecosystem longevity [81]. While the global spotlight shines upon global restoration efforts, wider global-scale patterns of seed-disperser decline and ensuring that restoring species to a level where they can effectively contribute, ensuring areas and ecosystems targeted for restoration can sustain themselves through effective seed dispersal and recruitment is critical [82]. Accounting for wider global-scale patterns of seed-disperser decline and ensuring that restoring species to a level where they can effectively contribute to dispersal is a cornerstone of effective long-term restoration. This is especially key in overexploited and predominantly animal-dispersed ecosystems across the tropics [6,18,83]. Ultimately, while the immediate and most easily detected impact of overexploitation will probably be the downsizing of vertebrate communities, the disproportionate decreases in seed-dispersal distances will have lasting effects on community resilience and persistence.

## Supplementary Material



## Data Availability

Data are publicly available and from published sources referenced in the manuscript. All code availability and outputs are publicly available from [84]. Supplementary material is available online [85].
