# Supplementary material for: Overexploitation risks disproportionate vertebrate downsizing and declines in potential dispersal distances in frugivory networks
Source: Proc Biol Sci. 2026 Oct 28;293(2081):20260578. doi: 10.1098/rspb.2026.0578 (PMC13725531; doi:10.1098/rspb.2026.0578)
Supplement: Supplementary file 1 [file rspb.2026.0578.zip › rspb.2026.0578/rspb20260578_si_001.pdf]

Supplementary Materials

Overexploitation risks disproportionate vertebrate downsizing and declines in potential dispersal distances in frugivory networks

(10.1098/rspb.2026.0578)

Oscar Morton[1,2,3]*, Miguel Lurgi[4,5], & David P. Edwards[2,3]

[1]School of Biosciences, University of Sheffield, Sheffield, UK

[2]Department of Plant Sciences and Centre for Global Wood Security, University of Cambridge, Cambridge, UK

[3]Conservation Research Institute, University of Cambridge, Cambridge, UK

[4]Department of Biosciences. Swansea University, Singleton Park, Swansea, UK

[5]Theoretical Sciences Visiting Program (TSVP). Okinawa Institute of Science and Technology Graduate University, Onna, Japan

Corresponding author (o.morton@sheffield.ac.uk)

Data availability

All data is publicly available and from published sources referenced in the manuscript.

Code availability

All code is publicly available from https://github.com/OMorton/ExploitedEcologicalNetworks and 10.5281/zenodo.21033719.

Acknowledgements

DPE acknowledges support from the Natural Environment Research Council (NE/Z50404X/1) and OM received financial support from the Leverhulme Trust (ECF-2024-005).

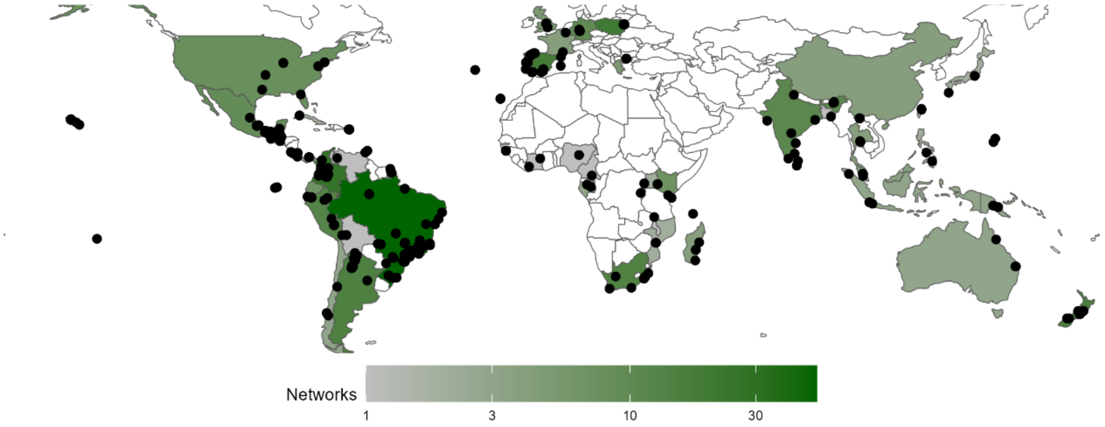

Supplementary Figure 1. Locations of all plant-frugivore networks. Data from Fricke et al. 2020.

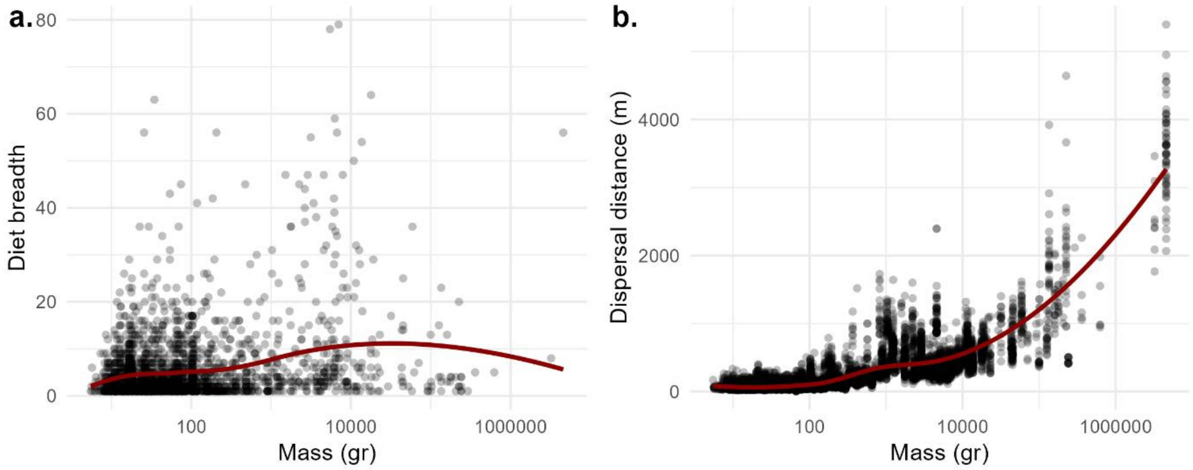

Supplementary Figure 2. Correlations between body mass and diet breadth and dispersal distance. Note in both plots each species may be represented by more than one point, as a given species if it appears in multiple networks, may slightly differ in diet breadth. Likewise, dispersal distance is interaction-specific so for each vertebrate species, there is a point per interaction. The red line is simply a locally estimated scatterplot smoothing line to ease visualisation. Body mass was weakly correlated with diet breadth (Spearman's rank correlation rho = 0.17, $p < 0.001$), and more clearly correlated with dispersal distance (Spearman's rank correlation rho = 0.78, $p < 0.001$). We note that the correlation between body mass and exploitation or trade has been previously discussed and shown in Scheffers et al., 2019 Science and Morton et al., 2023 Conservation Biology).

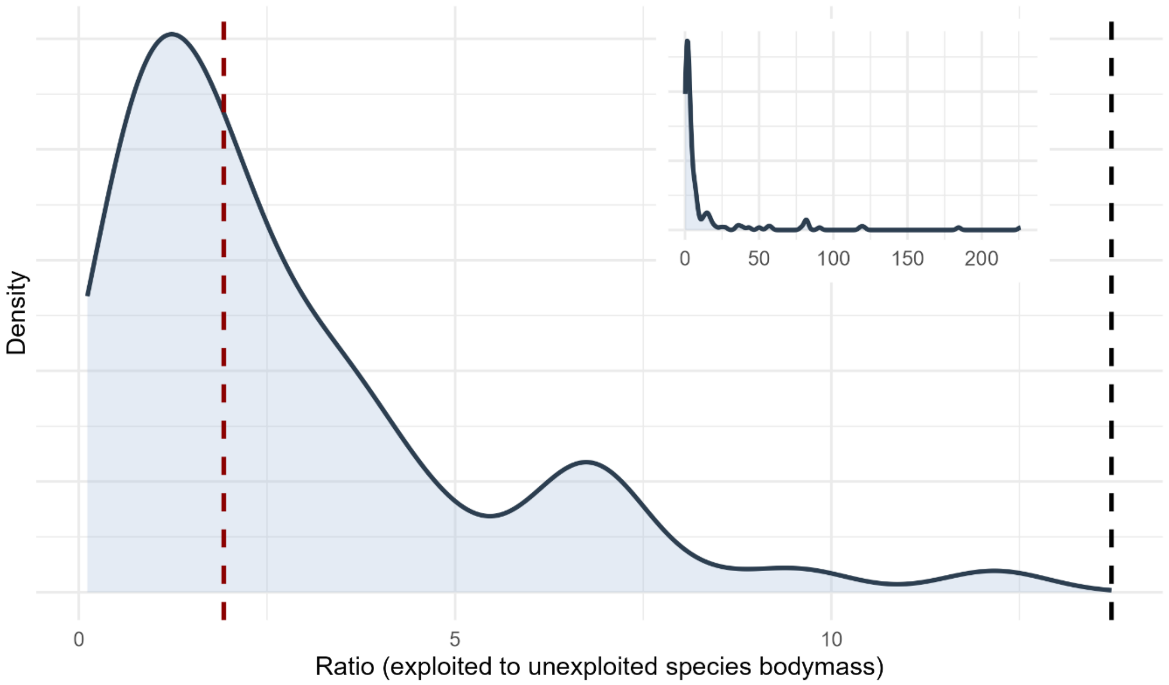

Supplementary Figure 3. Density plot of the ratio of average exploited and unexploited species body mass. The inset plot shows the full distribution of data. The distribution is driven primarily by a small number of networks containing a very large species (such as *Loxodonta africana*). The main plot shows the distribution containing the lowest 80% of observed ratios. The black and red dashed lines indicate the median body mass ratio of exploited vs unexploited species across the whole dataset (13.7) and for the lowest 80% of the data (1.9), respectively.

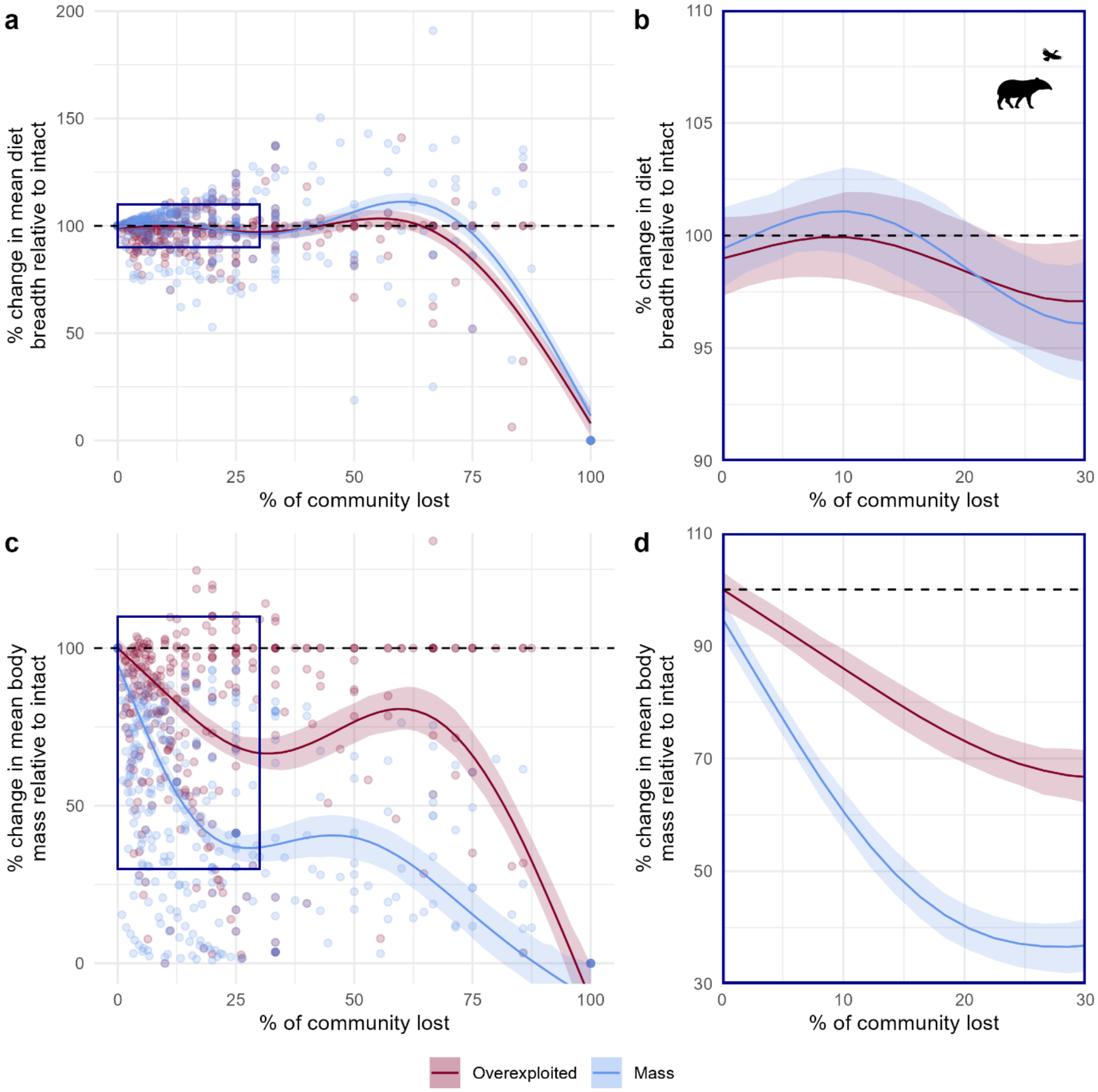

Supplementary Figure 4. Vertebrate community structural disruption following the local loss of likely overexploited species from local frugivory networks compared to a size structured null. (a) percentage change in mean community frugivore diet breadth after the loss of overexploited and randomly sampled species, the blue bounding box highlights the losses up to 30% of the intact community richness which is enlarged for ease of visualisation in (b). (c) and (d) show the same but for the change in community average body mass post extirpation relative to the intact community. Lines are posterior medians and the ribbons the 95% highest density interval.

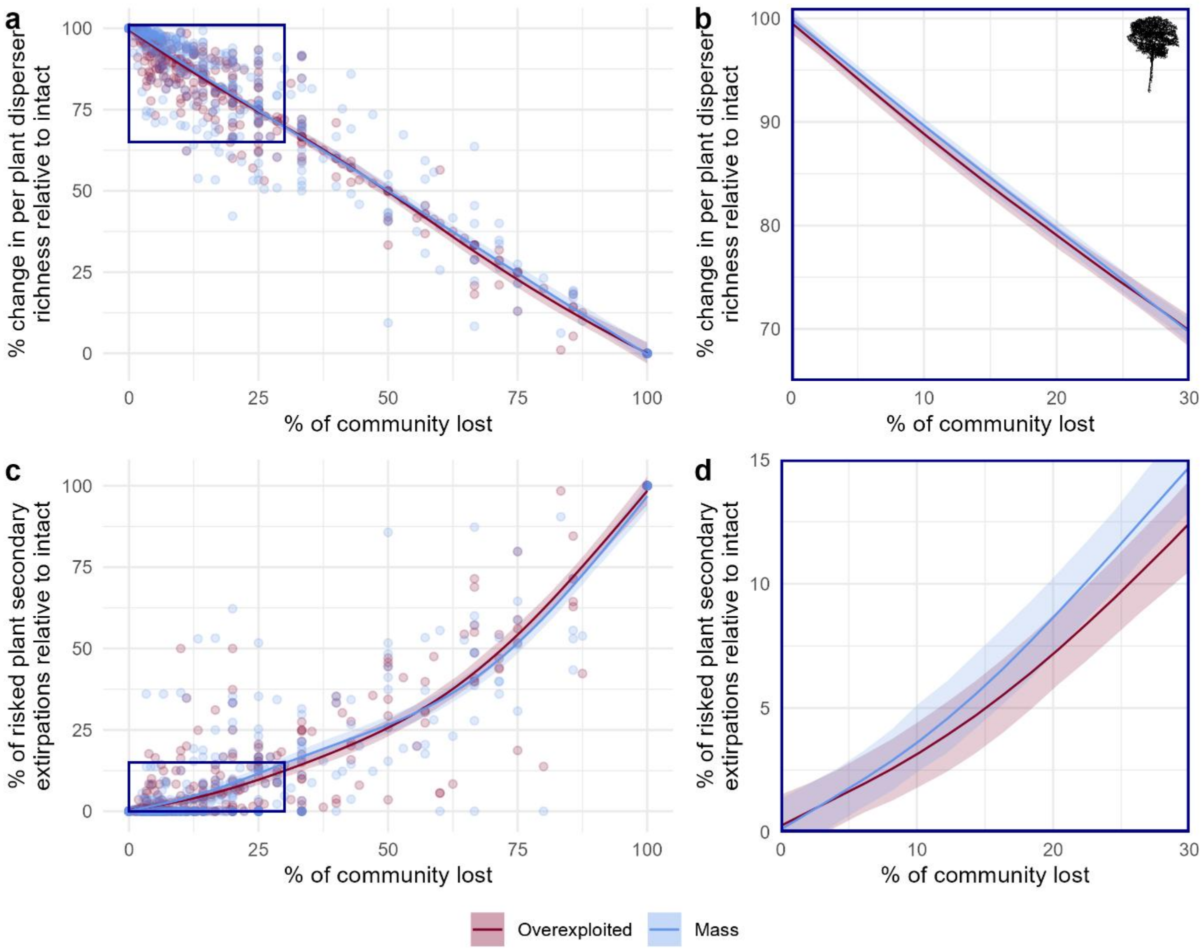

Supplementary Figure 5. Plant community structural disruption following the local loss of likely overexploited species from local frugivory networks compared to a size structured null. (a) percentage change in mean potential dispersers per plant species after the loss of overexploited and randomly sampled species (null expectation), the blue bounding box highlights the losses up to 30% of the intact community richness which is enlarged for ease of visualisation in (c). (d) and (e) show the same but for the number of risked structural secondary extirpations relative to the intact community richness. Lines are posterior medians and the ribbons the 95% highest density interval.

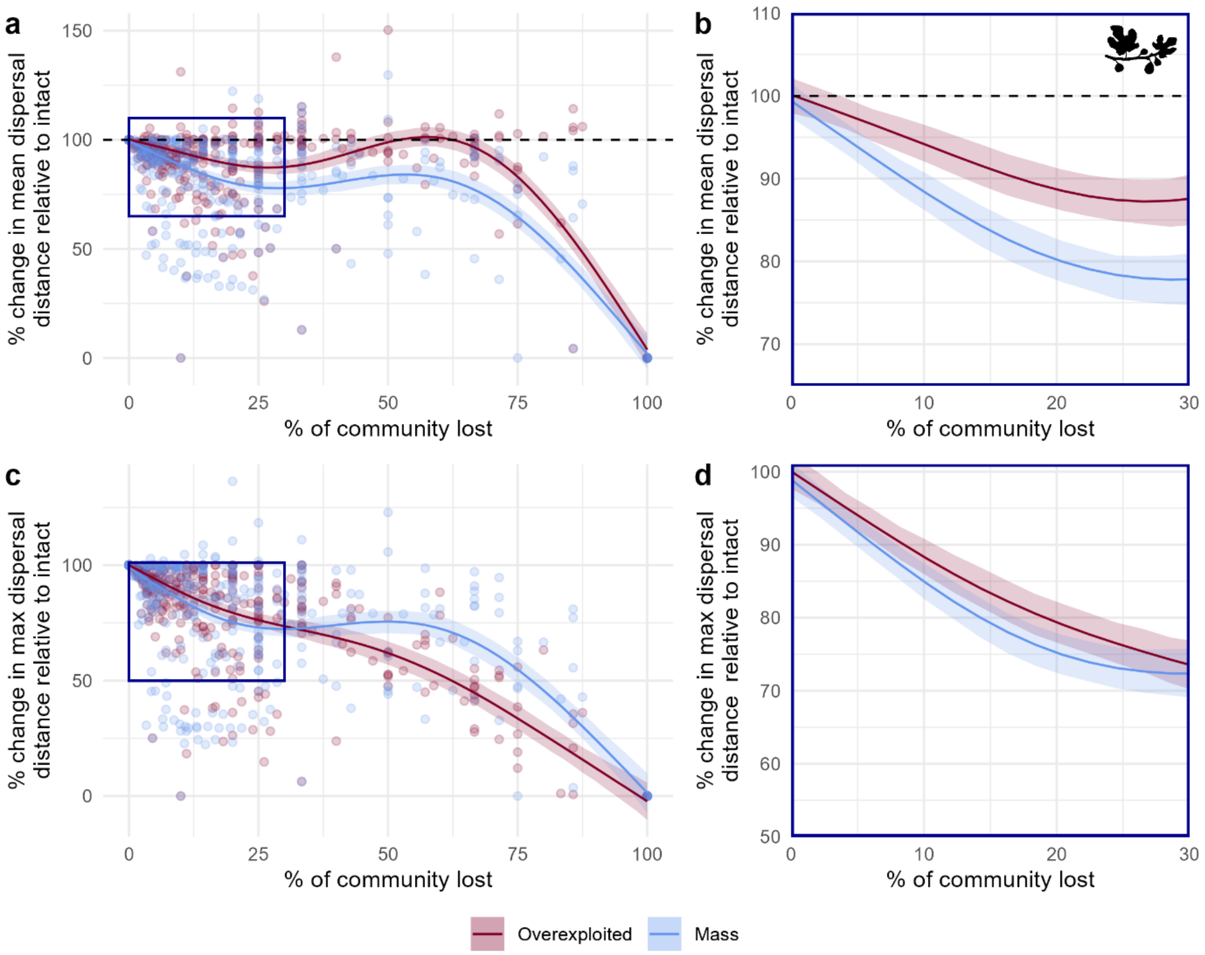

Supplementary Figure 6. Seed dispersal functioning disruption following the local loss of likely overexploited species from local frugivory networks compared to a size structured null. (a) percentage change in mean dispersal distance per network after the loss of overexploited and randomly sampled species (null expectation), the blue bounding box highlights the losses up to 30% of the intact community richness which is enlarged for ease of visualisation in (c). (d) and (e) show the same but percentage change in the community average maximum dispersal distance per plant species. Lines are posterior medians and the ribbons the 95% highest density interval.

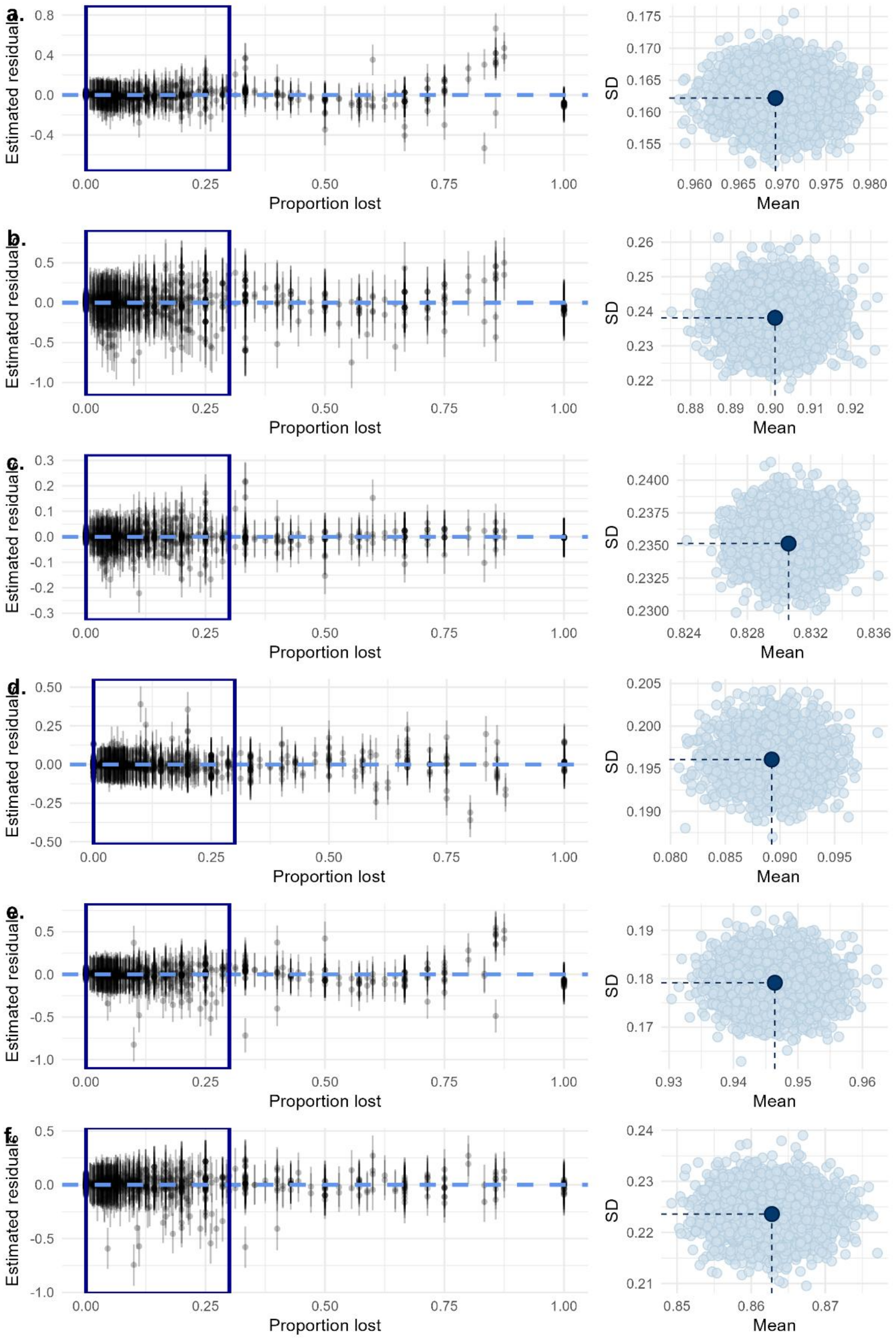

Supplementary Figure 7. Summary of model fitting. Each row shows the posterior residual and 95% HDI plotted against the proportion of species lost, and then a simple summary of the model's ability to recover the observed mean and SD of the observed data. We note a good fit across all modes, particularly up to the loss of 30% of species, where the majority of results are inferred from. Panels denote species models: average frugivore diet breadth (a), average body mass (b), average plant disperser richness (c), number of structural secondary extinctions (d), mean seed dispersal distance (e), and maximum seed dispersal distance (f).

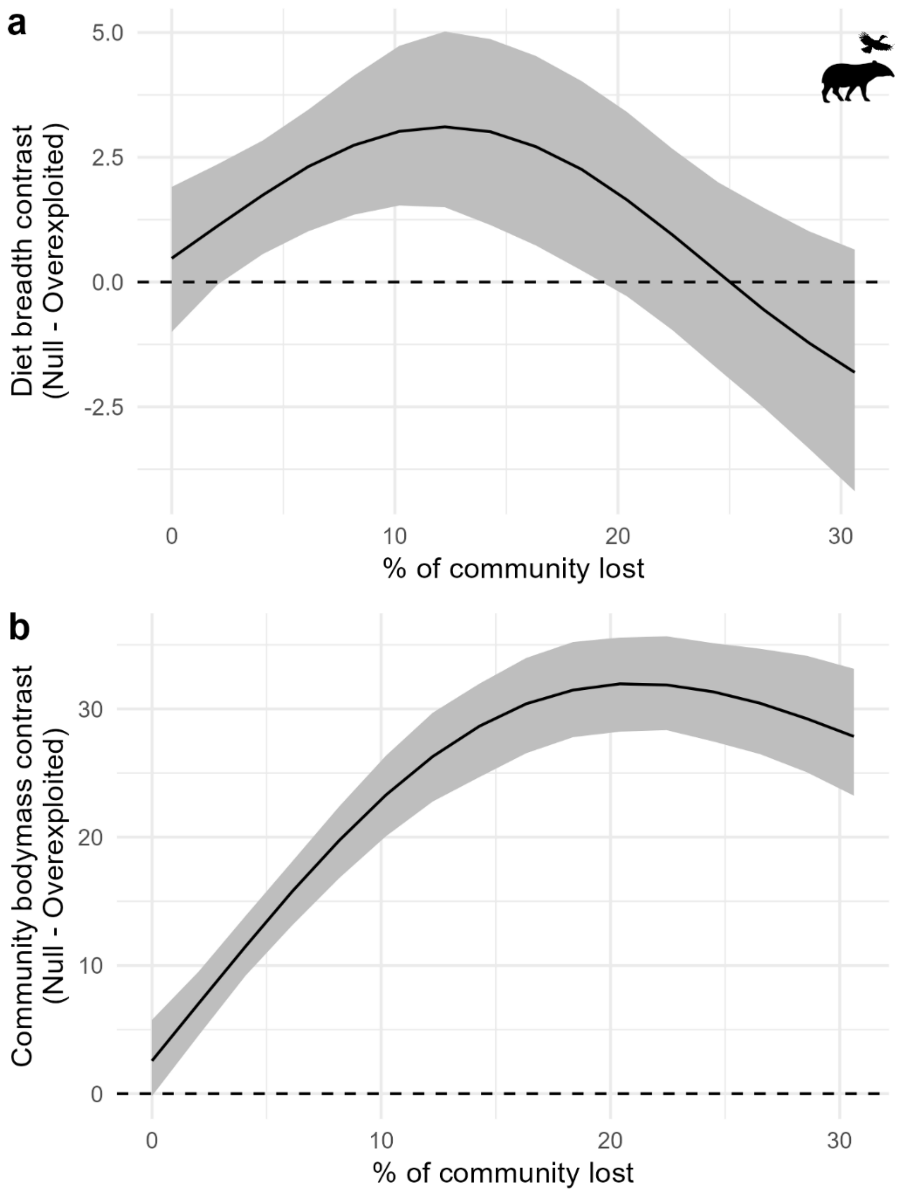

Supplementary Figure 8. Contrasted null and overexploitation-based extirpation scenarios for vertebrate community structure. (a) Contrasted diet breadth and (b) community body mass based on the results presented in Figure 2. Lines are posterior medians and the ribbons the 95% highest density interval.

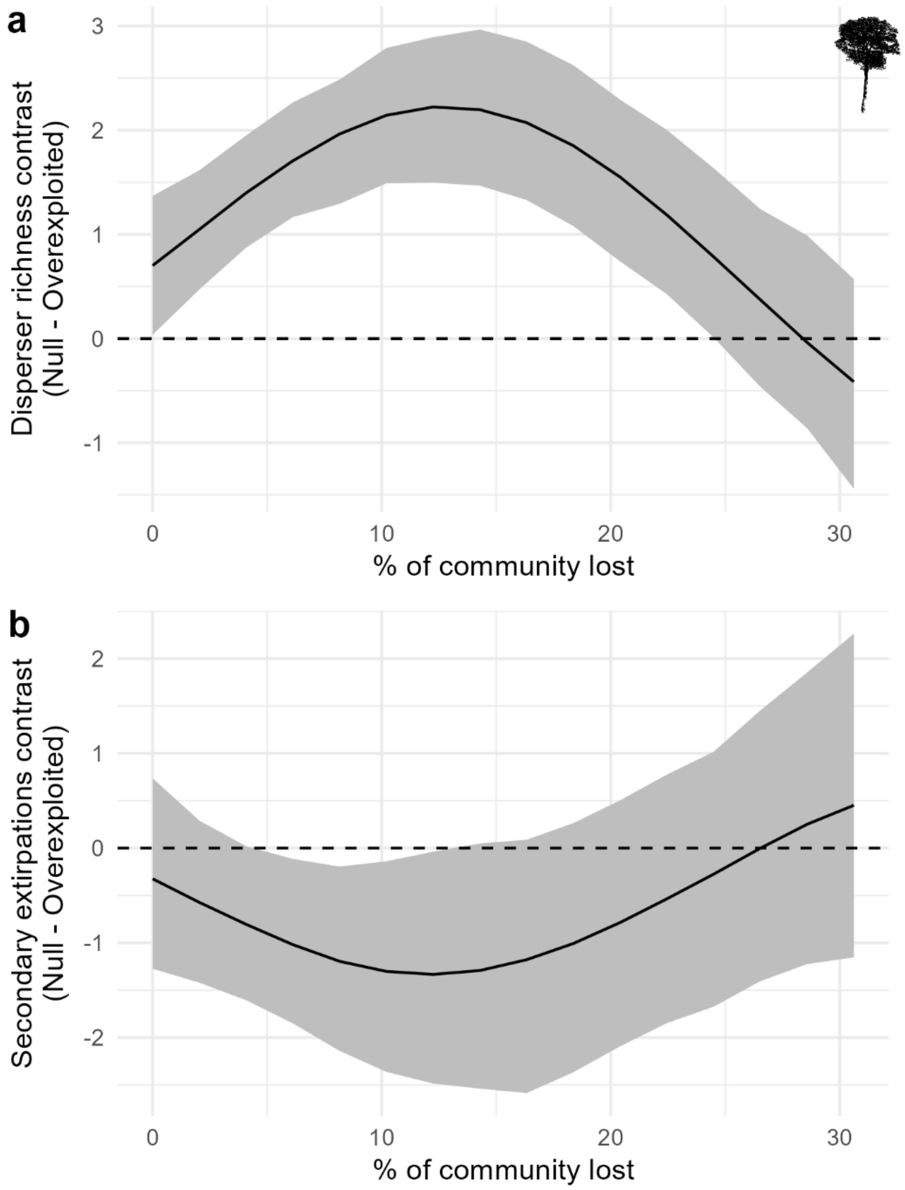

Supplementary Figure 9. Contrasted null and overexploitation-based extirpation scenarios for plant community structure. (a) Contrasted disperser richness and (b) secondary extirpations, based on the results presented in Figure 3. Lines are posterior medians and the ribbons the 95% highest density interval.

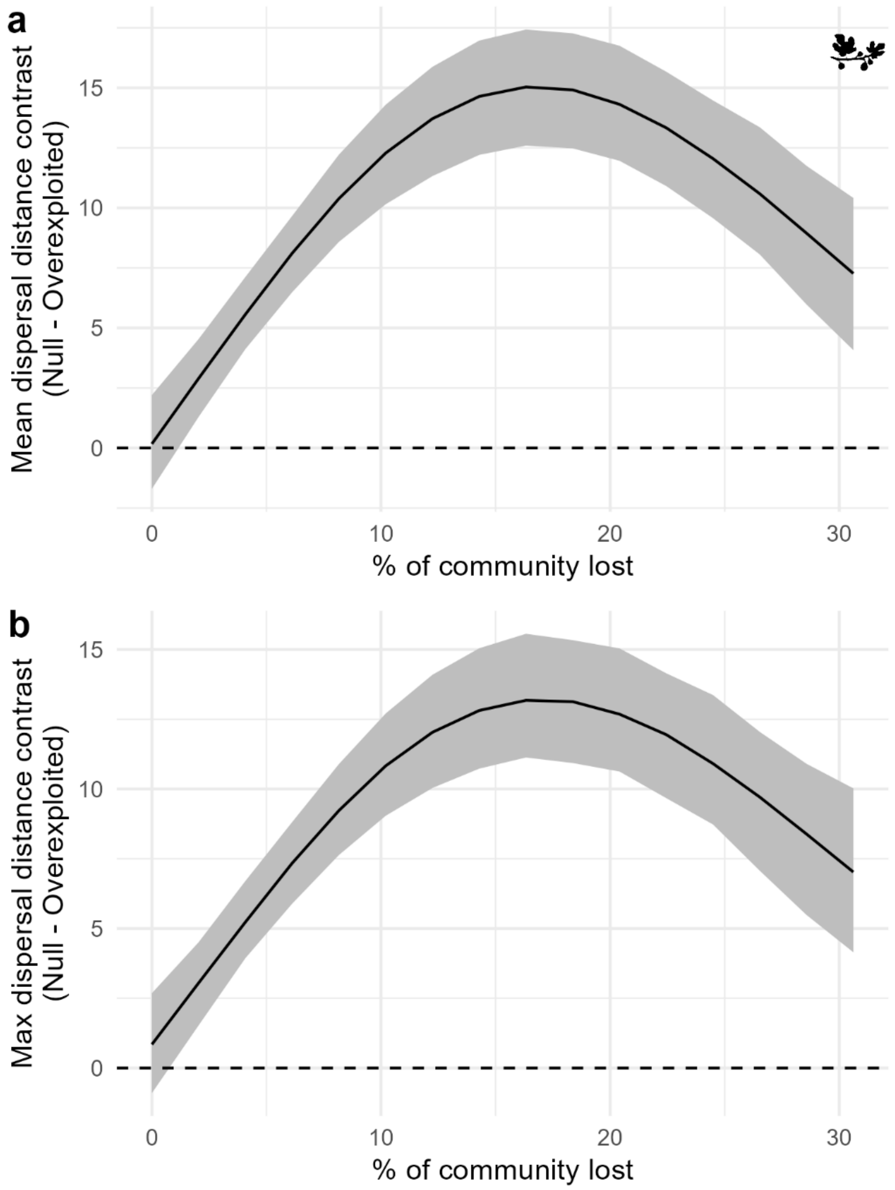

Supplementary Figure 10. Contrasted null and overexploitation-based extirpation scenarios for seed dispersal functioning. (a) Contrasted mean dispersal distance and (b) maximum dispersal distance, based on the results presented in Figure 4. Lines are posterior medians and the ribbons the 95% highest density interval.

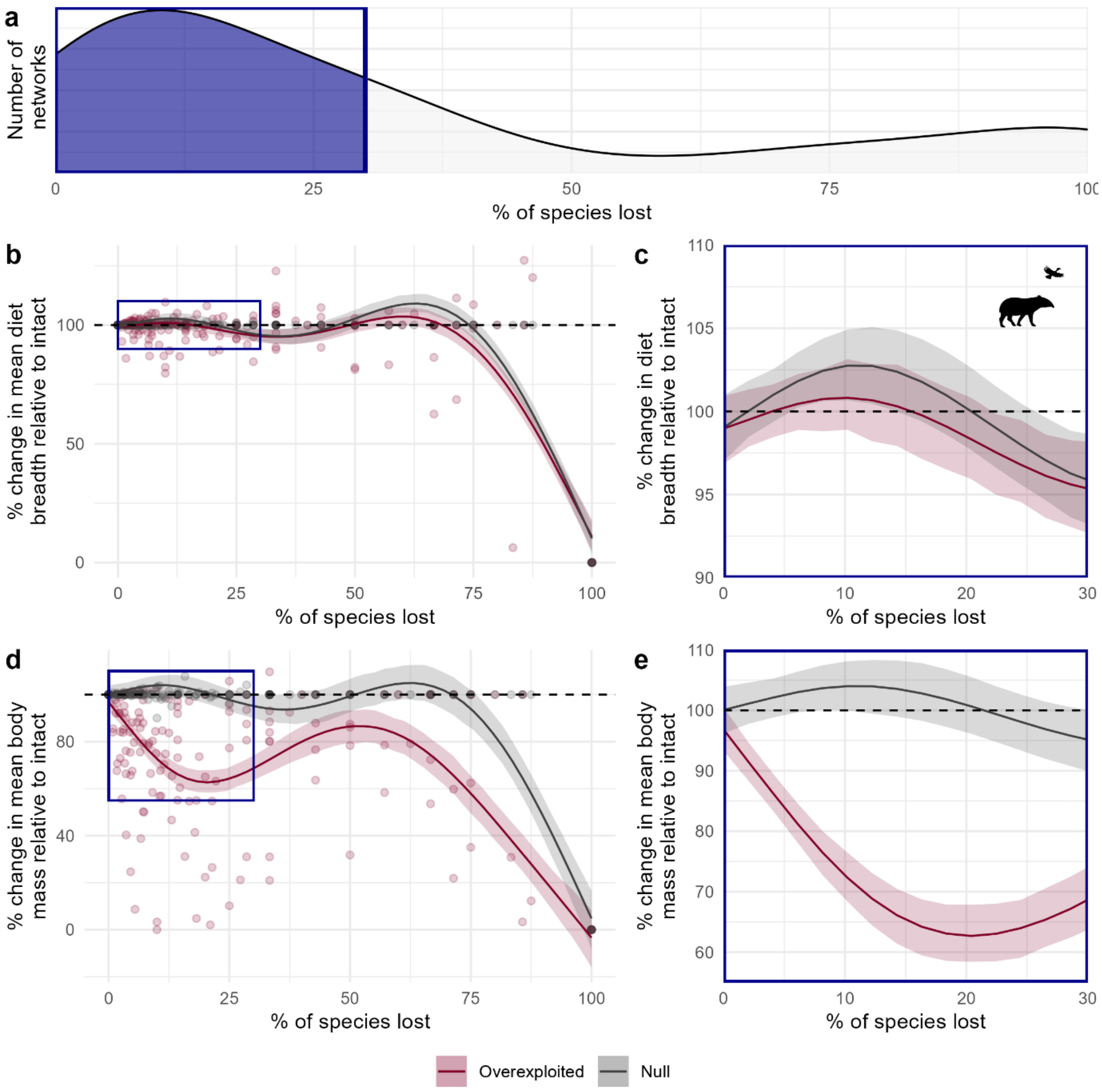

Supplementary Figure 11. Vertebrate community structural disruption following the local loss of likely overexploited species from local frugivory networks (excluding the loss of Least Concern species). (a) The distribution of exploited species proportionate presence in 160 frugivory networks, blue bounding box shows 80% of all networks contain 30% or less overexploited species (null expectation), (b) percentage change in mean community frugivore diet breadth after the loss of overexploited and randomly sampled species, the blue bounding box highlights the losses up to 30% of the intact community richness which is enlarged for ease of visualisation in (c). (d) and (e) show the same but for the change in community average body mass post extirpation relative to the intact community. Lines are posterior medians and the ribbons the 95% highest density interval.

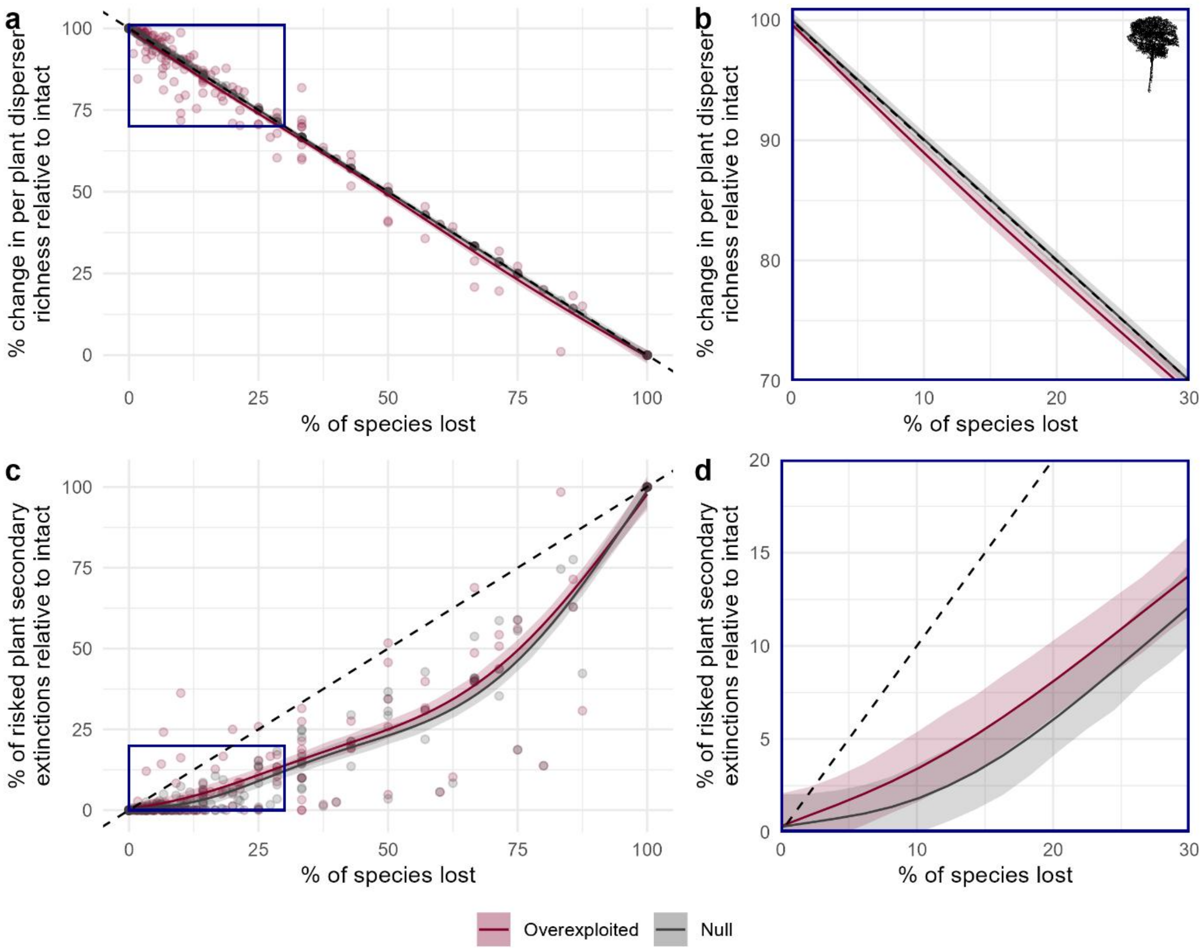

Supplementary Figure 12. Plant community structural disruption following the local loss of likely overexploited species from local frugivory networks (excluding the loss of Least Concern species). (a) percentage change in mean potential dispersers per plant species after the loss of overexploited and randomly sampled species (null expectation), the blue bounding box highlights the losses up to 30% of the intact community richness which is enlarged for ease of visualisation in (c). (d) and (e) show the same but for the number of risked structural secondary extirpations relative to the intact community richness. Lines are posterior medians and the ribbons the 95% highest density interval.

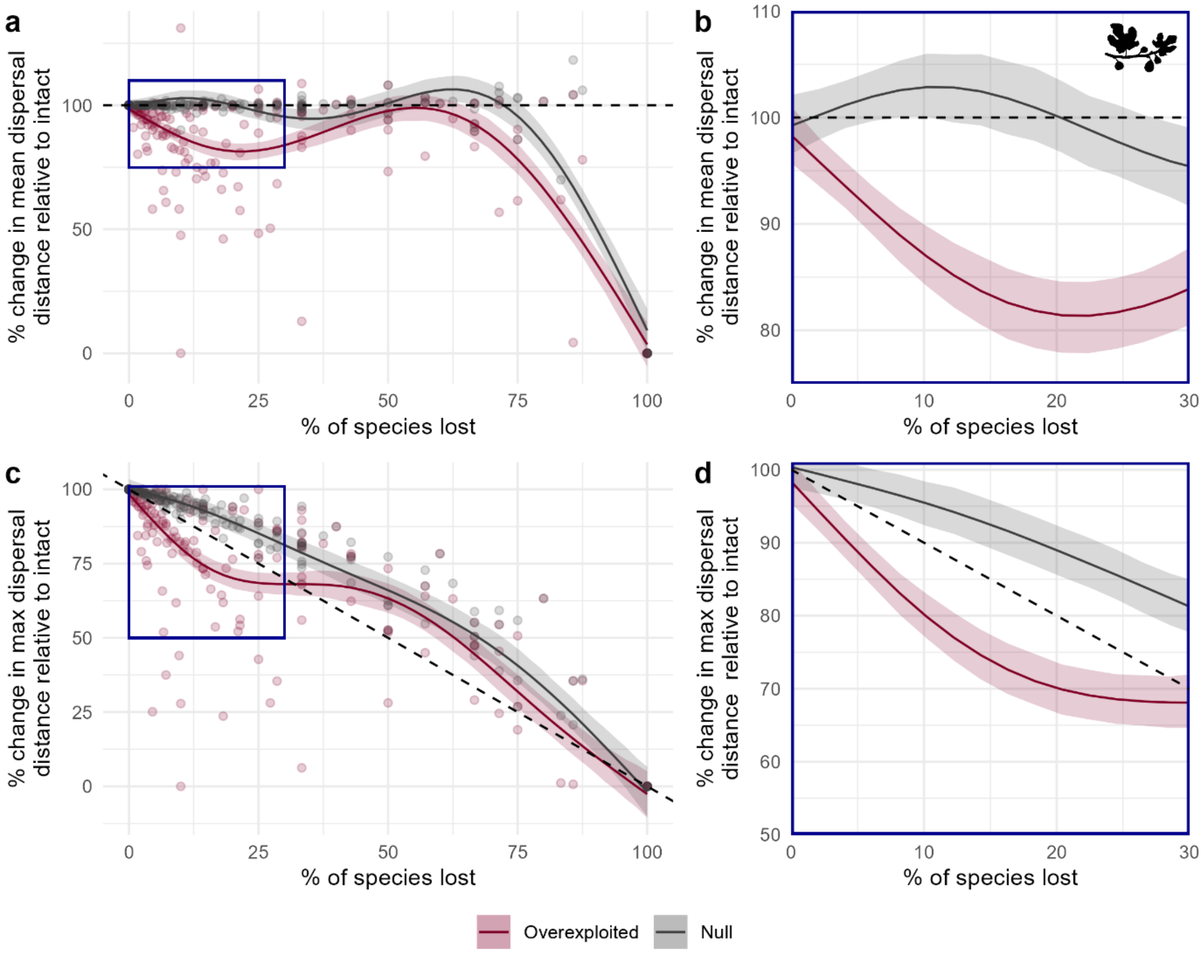

Supplementary Figure 13. Seed dispersal functioning disruption following the local loss of likely overexploited species from local frugivory networks (excluding the loss of Least Concern species). (a) percentage change in mean dispersal distance per network after the loss of overexploited and randomly sampled species (null expectation), the blue bounding box highlights the losses up to 30% of the intact community richness which is enlarged for ease of visualisation in (c). (d) and (e) show the same but percentage change in the community average maximum dispersal distance per plant species. Lines are posterior medians and the ribbons the 95% highest density interval.

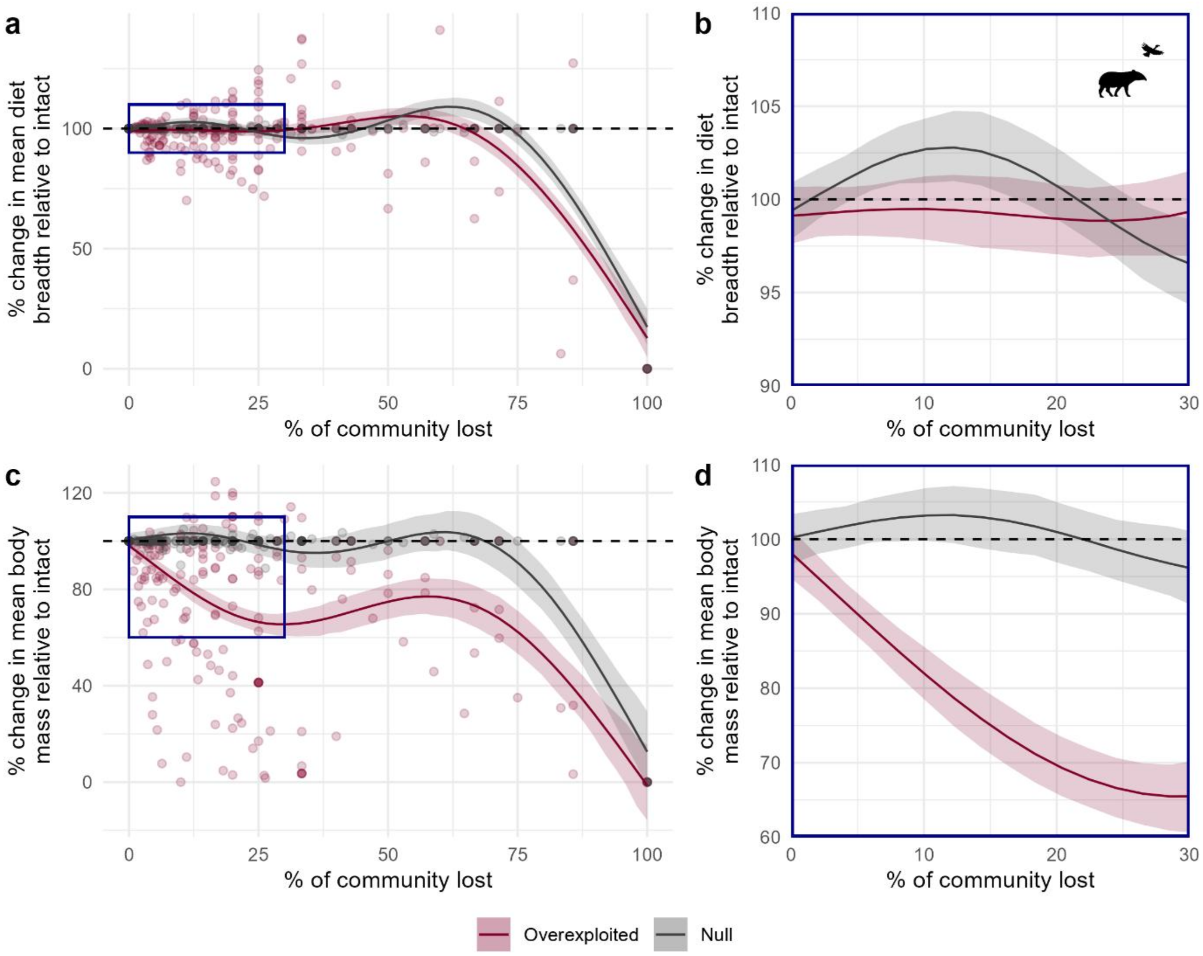

Supplementary Figure 14. Vertebrate community structural disruption following the local loss of likely overexploited species from local frugivory networks (only for networks where birds and mammals were comprehensively sampled). (a) The distribution of exploited species proportionate presence in 160 frugivory networks, blue bounding box shows 80% of all networks contain 30% or less overexploited species (null expectation), (b) percentage change in mean community frugivore diet breadth after the loss of overexploited and randomly sampled species, the blue bounding box highlights the losses up to 30% of the intact community richness which is enlarged for ease of visualisation in (c). (d) and (e) show the same but for the change in community average body mass post extirpation relative to the intact community. Lines are posterior medians and the ribbons the 95% highest density interval.

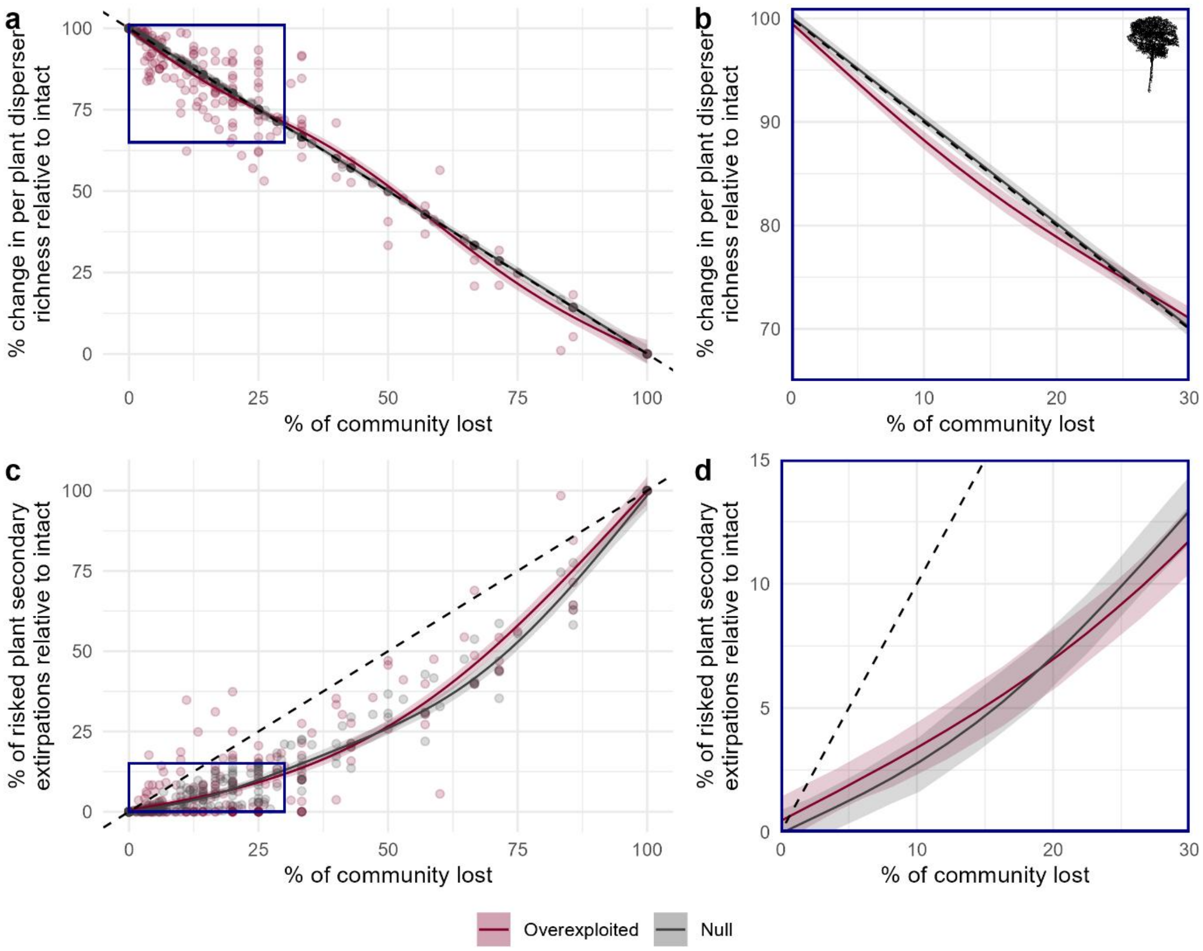

Supplementary Figure 15. Plant community structural disruption following the local loss of likely overexploited species from local frugivory networks (only for networks where birds and mammals were comprehensively sampled). (a) percentage change in mean potential dispersers per plant species after the loss of overexploited and randomly sampled species (null expectation), the blue bounding box highlights the losses up to 30% of the intact community richness which is enlarged for ease of visualisation in (c). (d) and (e) show the same but for the number of risked structural secondary extirpations relative to the intact community richness. Lines are posterior medians and the ribbons the 95% highest density interval.

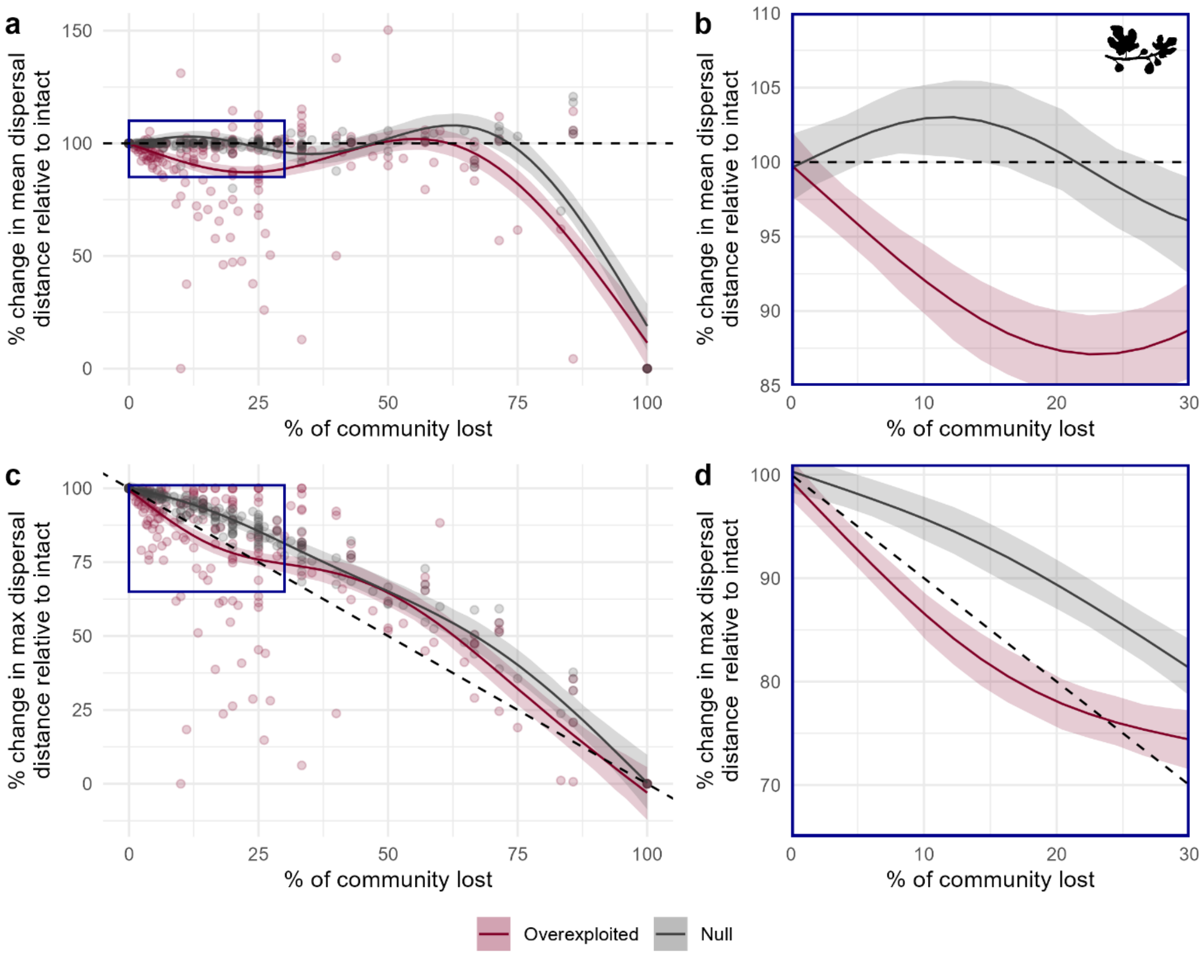

Supplementary Figure 16. Seed dispersal functioning disruption following the local loss of likely overexploited species from local frugivory networks (only for networks where birds and mammals were comprehensively sampled). (a) percentage change in mean dispersal distance per network after the loss of overexploited and randomly sampled species (null expectation), the blue bounding box highlights the losses up to 30% of the intact community richness which is enlarged for ease of visualisation in (c). (d) and (e) show the same but percentage change in the community average maximum dispersal distance per plant species. Lines are posterior medians and the ribbons the 95% highest density interval.

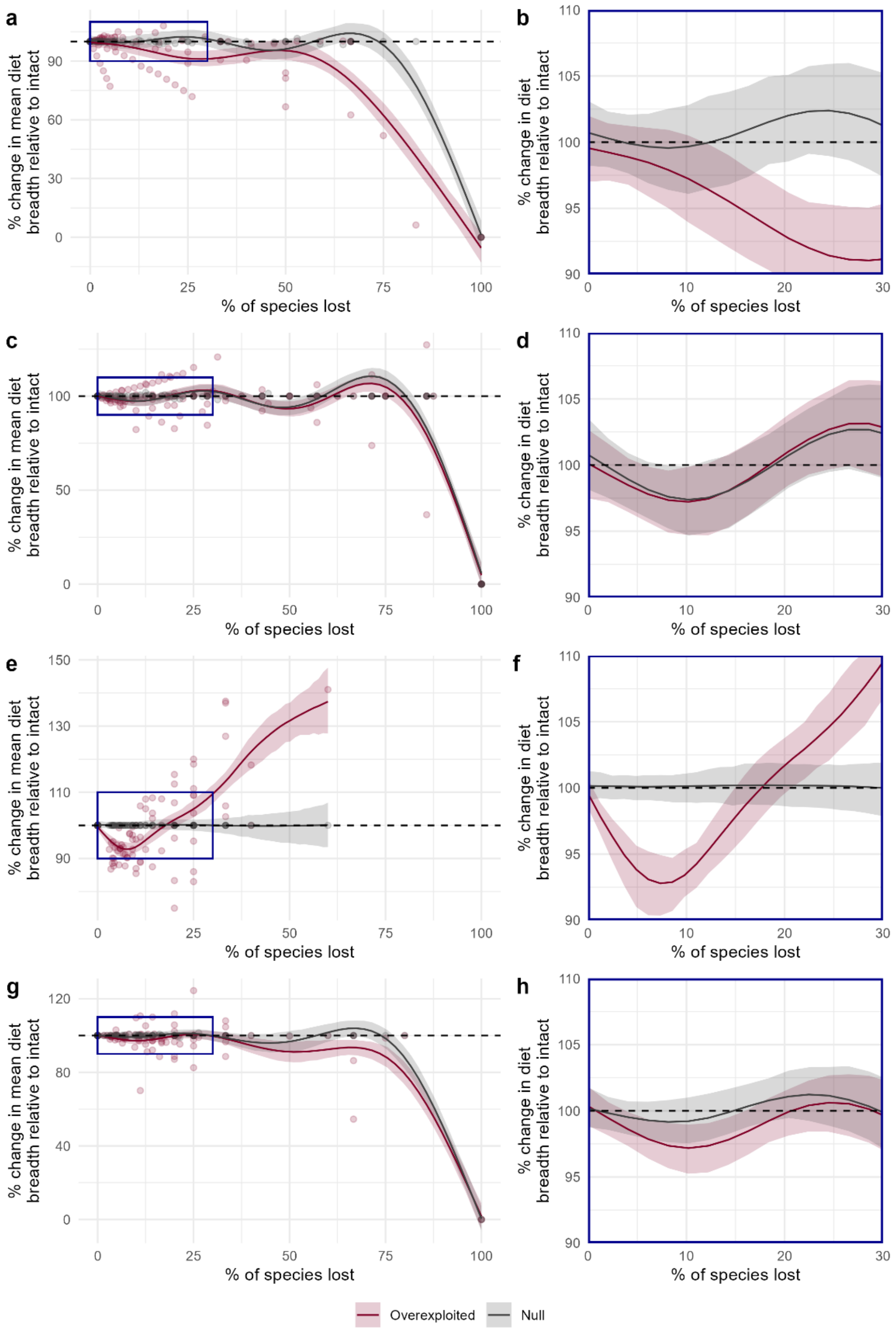

Supplementary Figure 17. Regional vertebrate community percentage change in mean community frugivore diet breadth following the local loss of likely overexploited species from local frugivory networks. (a) percentage change in mean community frugivore diet breadth after the loss of overexploited and randomly sampled species across the Americas, the blue bounding box highlights the losses up to 30% of the intact community richness which is enlarged for ease of visualisation in (b). (c) and (d) show the same for Africa, (e) and (f) for Eurasia, and (g) and (h) for Asia-Pacific. Lines are posterior medians and the ribbons the 95% highest density interval.

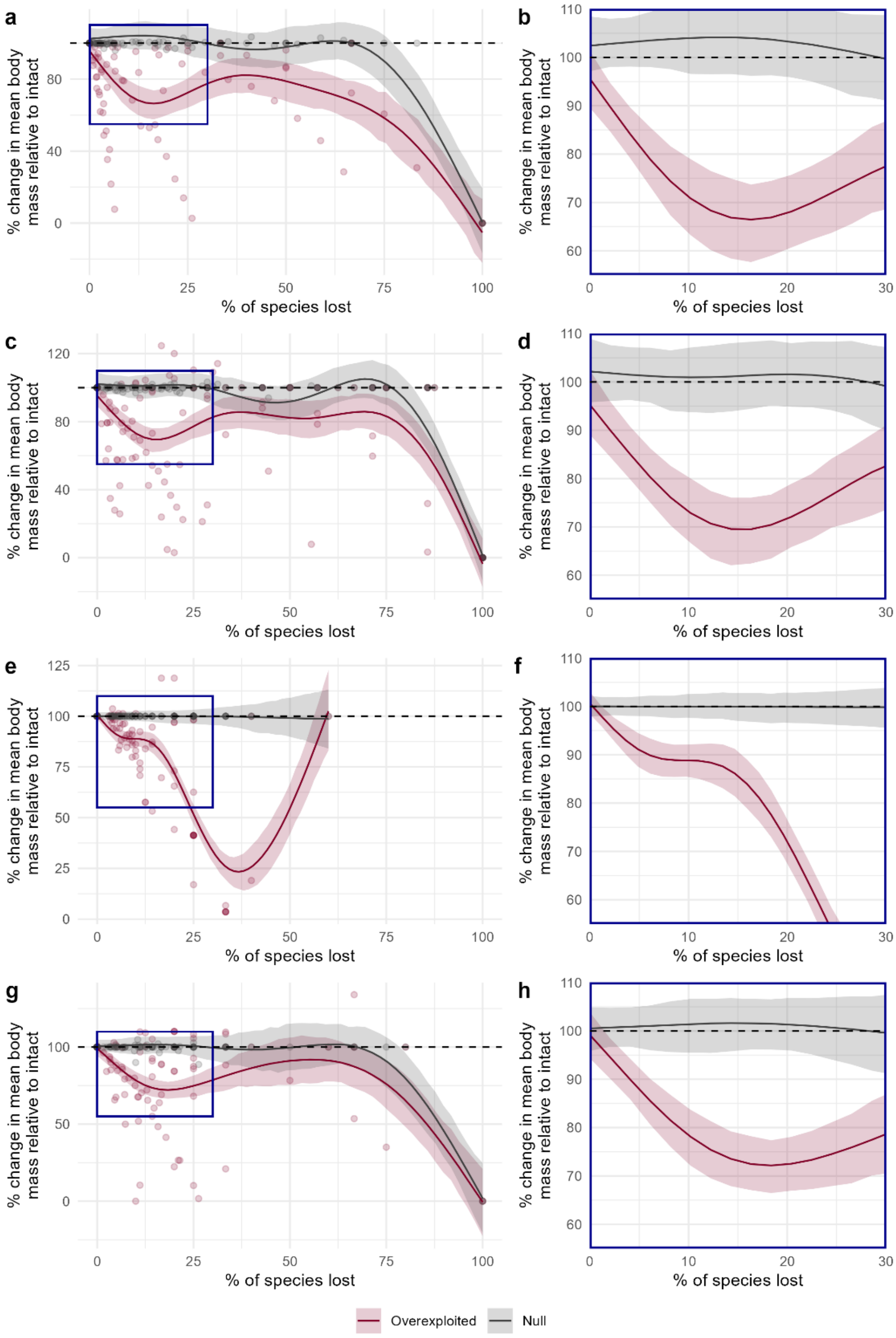

Supplementary Figure 18. Regional vertebrate community percentage change in community average body mass following the local loss of likely overexploited species from local frugivory networks. (a) percentage change in mean community frugivore diet breadth after the loss of overexploited and randomly sampled species across the Americas, the blue bounding box highlights the losses up to 30% of the intact community richness which is enlarged for ease of visualisation in (b). (c) and (d) show the same for Africa, (e) and (f) for Eurasia, and (g) and (h) for Asia-Pacific. Lines are posterior medians and the ribbons the 95% highest density interval.

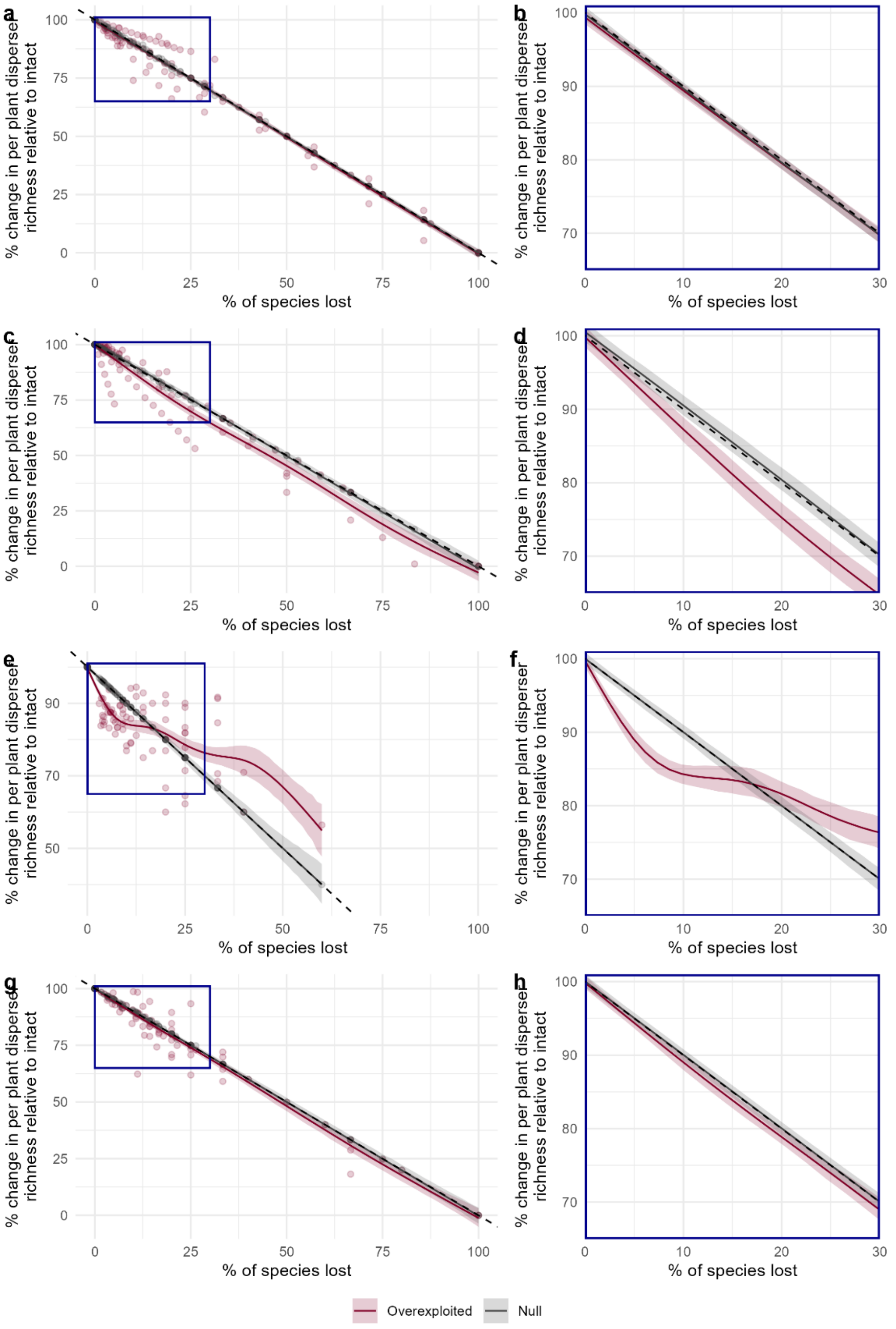

Supplementary Figure 19. Regional plant community percentage change in mean potential dispersers per plant species following the local loss of likely overexploited species from local frugivory networks. (a) percentage change in mean community frugivore diet breadth after the loss of overexploited and randomly sampled species across the Americas, the blue bounding box highlights the losses up to 30% of the intact community richness which is enlarged for ease of visualisation in (b). (c) and (d) show the same for Africa, (e) and (f) for Eurasia, and (g) and (h) for Asia-Pacific. Lines are posterior medians and the ribbons the 95% highest density interval.

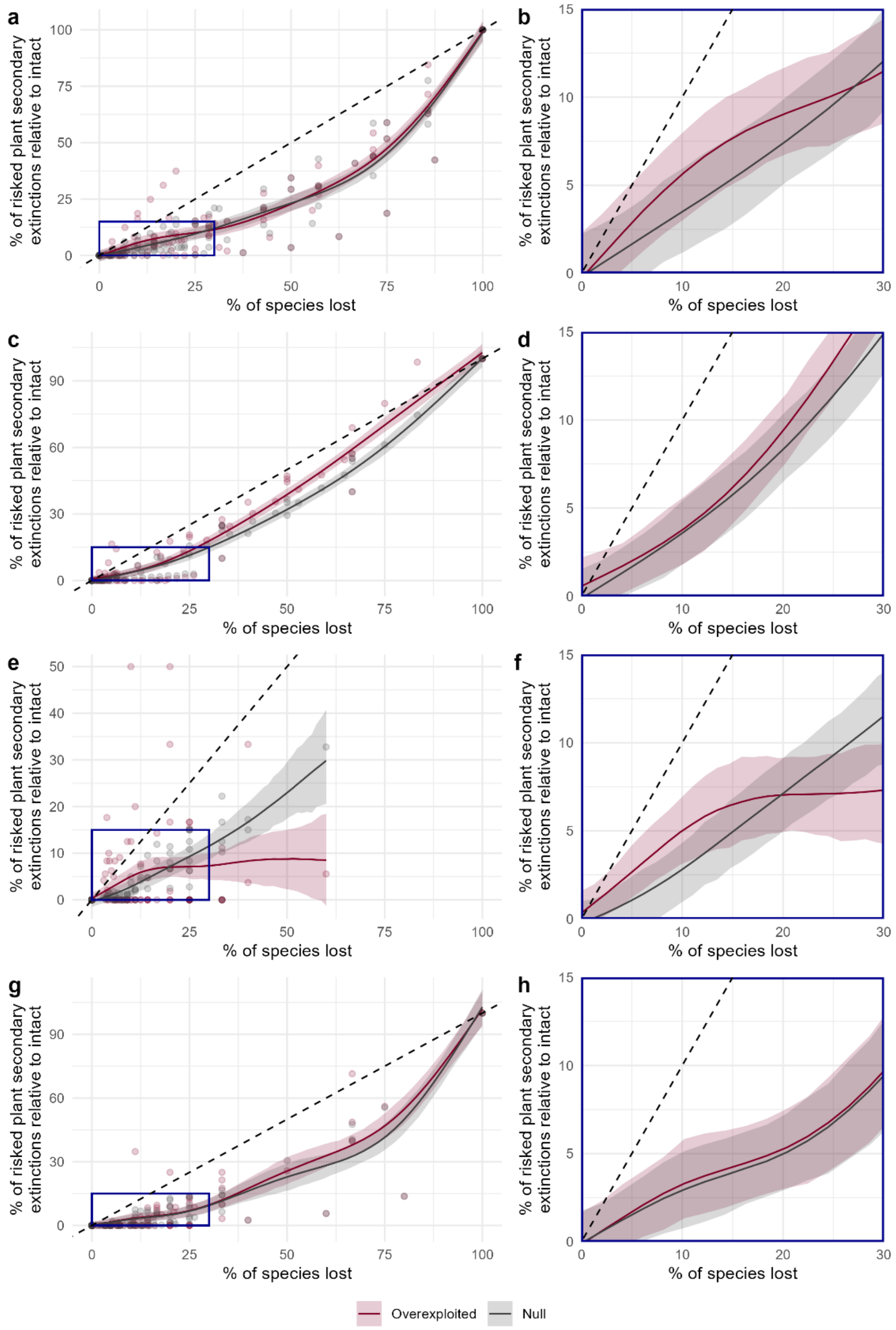

Supplementary Figure 20. Regional plant community risked structural secondary extirpations relative to the intact community richness following the local loss of likely overexploited species from local frugivory networks. (a) percentage change in mean community frugivore diet breadth after the loss of overexploited and randomly sampled species across the Americas, the blue bounding box highlights the losses up to 30% of the intact community richness which is enlarged for ease of visualisation in (b). (c) and (d) show the same for Africa, (e) and (f) for Eurasia, and (g) and (h) for Asia-Pacific. Lines are posterior medians and the ribbons the 95% highest density interval.

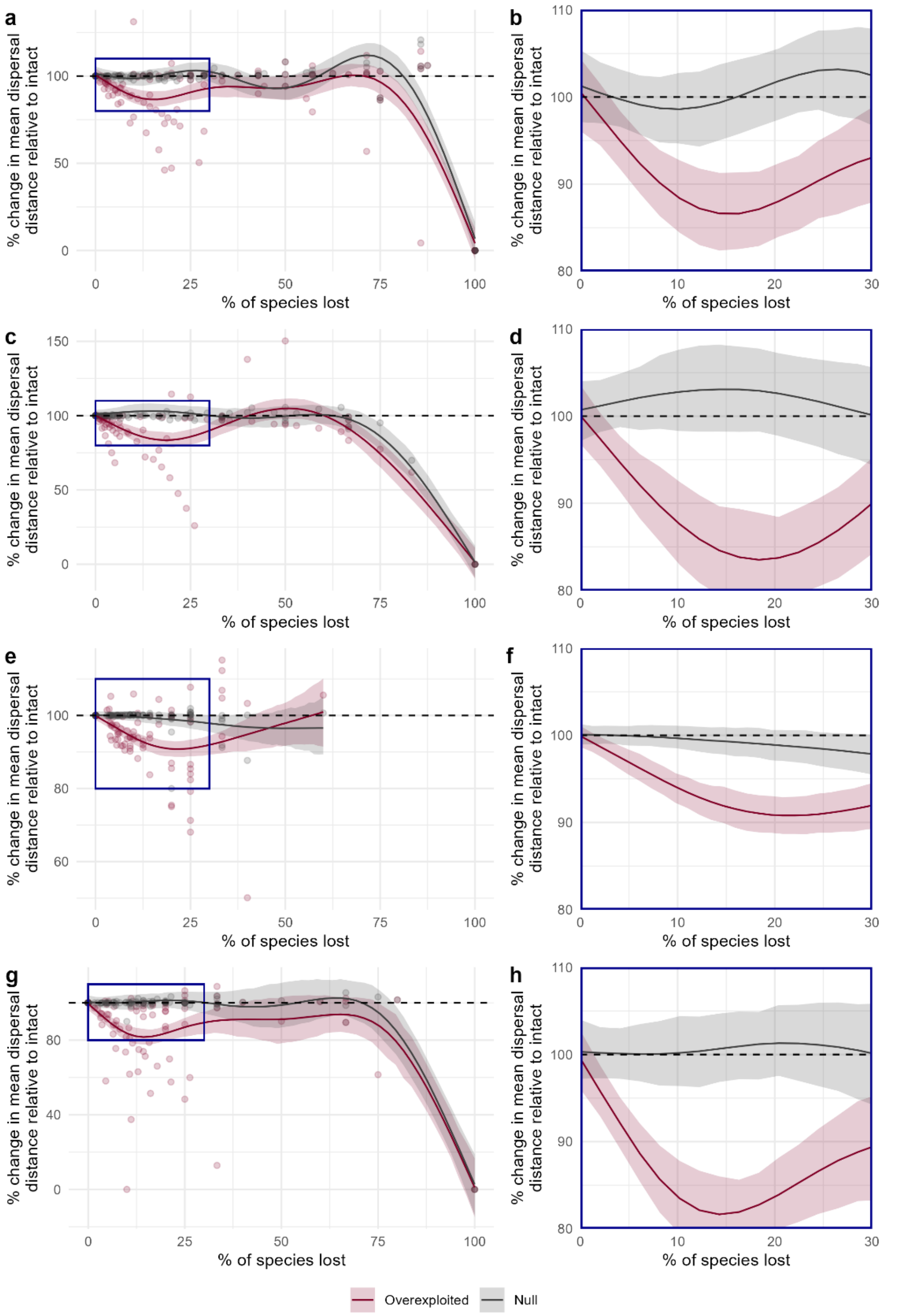

Supplementary Figure 21. Regional percentage change in mean dispersal distance per network after the loss of likely overexploited species from local frugivory networks. (a) percentage change in mean community frugivore diet breadth after the loss of overexploited and randomly sampled species across the Americas, the blue bounding box highlights the losses up to 30% of the intact community richness which is enlarged for ease of visualisation in (b). (c) and (d) show the same for Africa, (e) and (f) for Eurasia, and (g) and (h) for Asia-Pacific. Lines are posterior medians and the ribbons the 95% highest density interval.

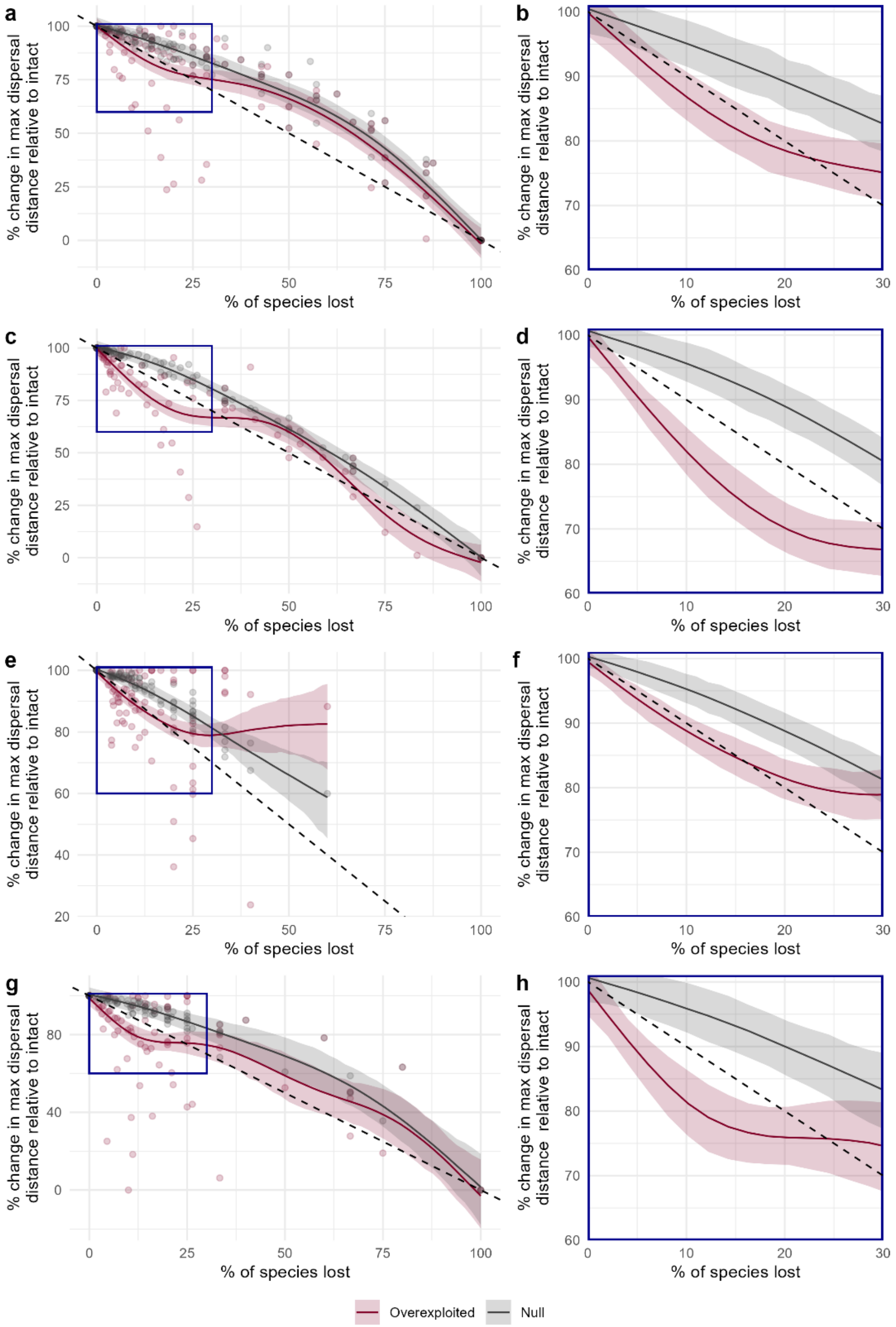

Supplementary Figure 22. Regional percentage change in community average maximum dispersal distance per plant species after the loss of likely overexploited species from local frugivory networks. (a) percentage change in mean community frugivore diet breadth after the loss of overexploited and randomly sampled species across the Americas, the blue bounding box highlights the losses up to 30% of the intact community richness which is enlarged for ease of visualisation in (b). (c) and (d) show the same for Africa, (e) and (f) for Eurasia, and (g) and (h) for Asia-Pacific. Lines are posterior medians and the ribbons the 95% highest density interval.

Supplementary Table 1. Scoring protocol applied to IUCN Threat Tables. Adapted from Marsh et al. 2021.

Category	Description	Score
**Timing**	Ongoing	3
	Unknown	2
	Past, likely to return	1
	Future	1
	Past, unlikely to return	-6
**Scope**	Whole (>90%)	3
	Majority (50-90%)	2
	Unknown	2
	Minority (<50%)	1
**Severity**	Very rapid declines	3
	Rapid declines	2
	Unknown	2
	Slow, Significant Declines	1
	Causing/Could cause fluctuations	1
	Negligible declines	0
	No decline	0

Supplementary Table 2. Priors used for fitting all models.

Coefficient	Distribution	Notes
Intercept	Normal(0,1)	
β	Normal(0,5	
SD	Student_t(3, 0, 2.5)	With a lower bound of 0
SD of the smooth	Student_t(3, 0, 2.5)	With a lower bound of 0
Sigma	Student_t(3, 0, 2.5)	With a lower bound of 0

Supplementary Table 3. All coefficients from the models presented in the main text. We note that, given the models are hierarchical GAMs, individual coefficients may not be directly interpretable in the manner intended by the reader, thus we also provide all the marginal estimates and uncertainties shown in the main text figures at https://github.com/OMorton/ExploitedEcologicalNetworks. In the table below, UCI and LCI denote the upper and lower 95% confidence intervals, and ESS denotes the effective sample size. The mean, confidence intervals and Rhat values are all shown to 2 decimal places. As described in the main text, each model follows the same structure - *~ 1 + Scenario + s(Proportion.Lost, By = scenario, bs = "tp", k = 5) + (1|network.id).*

Model	Parameter	Mean	95% LCI	95% UCI	Rhat	Bulk ESS	Tail ESS
**Diet breadth**	Intercept	0.96	0.95	0.97	1	4667	3073
	Scenario[Null]	0.02	0.01	0.03	1	6745	1900
	smooth(ProportionLost*Scenario[Exploited])	-4.73	-5.07	-4.38	1	5565	2259
	smooth(ProportionLost*Scenario[Null])	-5.16	-5.51	-4.81	1	6534	2494
**Average body mass**	Intercept	0.83	0.81	0.85	1	3278	2166
	Scenario[Null]	0.15	0.13	0.17	1	6237	2114
	smooth(ProportionLost*Scenario[Exploited])	-6.87	-7.55	-6.18	1	5287	2316
	smooth(ProportionLost*Scenario[Null])	-5.09	-5.78	-4.42	1	4393	2458
**Disperser richness**	Intercept	0.83	0.82	0.83	1	3145	2831
	Scenario[Null]	0.01	0.01	0.02	1.01	6175	2154
	smooth(ProportionLost*Scenario[Exploited])	-2.67	-2.83	-2.51	1	3463	2421
	smooth(ProportionLost*Scenario[Null])	-2.77	-2.88	-2.67	1	2779	2182
**Secondary extinctions**	Intercept	0.09	0.09	0.1	1	2173	2448
	Scenario[Null]	-0.01	-0.01	0	1	8241	1996
	smooth(ProportionLost*Scenario[Exploited])	3.04	2.79	3.3	1	2821	2129
	smooth(ProportionLost*Scenario[Null])	3.27	3.02	3.51	1	3407	2647
**Mean dispersal distance**	Intercept	0.92	0.91	0.93	1	3213	2667
	Scenario[Null]	0.06	0.05	0.07	1	6695	2297
	smooth(ProportionLost*Scenario[Exploited])	-5.97	-6.45	-5.5	1	4842	2218
	smooth(ProportionLost*Scenario[Null])	-5.04	-5.5	-4.59	1	5800	2278
**Max dispersal distance**	Intercept	0.84	0.83	0.85	1	2581	2566
	Scenario[Null]	0.06	0.04	0.07	1	7533	1929
	smooth(ProportionLost*Scenario[Exploited])	-3.85	-4.25	-3.43	1	3401	2488
	smooth(ProportionLost*Scenario[Null])	-3.07	-3.49	-2.65	1	2136	2193

Supplementary Table 4. Summary statistics across the 160 included networks.

Metric	Mean	Median	Range
Vertebrate richness	15.6	9	2 to 109
Plant richness	21.4	12	3 to 171
Likely overexploited vertebrates	2.07	1	1 to 14
Proportion of likely overexploited vertebrates	0.25	0.14	0.01 to 1
Body mass (g)	10986.11	45.00	5.5 to 4565000
